# Comparison of approaches for increasing affinity of affibody molecules for imaging of B7-H3: dimerization and affinity maturation

**DOI:** 10.1186/s41181-024-00261-3

**Published:** 2024-04-16

**Authors:** Maryam Oroujeni, Matilda Carlqvist, Eva Ryer, Anna Orlova, Vladimir Tolmachev, Fredrik Y. Frejd

**Affiliations:** 1 Department of Immunology, Genetics and Pathology, Uppsala University, Uppsala, 751 85 Sweden; 2 Affibody AB, Solna, 171 65 Sweden; 3 Department of Medicinal Chemistry, Uppsala University, Uppsala, 751 83 Sweden

**Keywords:** B7-H3, Affibody molecule, Dimerization, Affinity maturation, Technetium-99m (^99m^Tc), SKOV-3 xenograft, SPECT/CT imaging

## Abstract

**Background:**

Radionuclide molecular imaging can be used to visualize the expression levels of molecular targets. Affibody molecules, small and high affinity non-immunoglobulin scaffold-based proteins, have demonstrated promising properties as targeting vectors for radionuclide tumour imaging of different molecular targets. B7-H3 (CD276), an immune checkpoint protein belonging to the B7 family, is overexpressed in different types of human malignancies. Visualization of overexpression of B7-H3 in malignancies enables stratification of patients for personalized therapies. Affinity maturation of anti-B7-H3 Affibody molecules as an approach to improve the binding affinity and targeting properties was recently investigated. In this study, we tested the hypothesis that a dimeric format may be an alternative option to increase the apparent affinity of Affibody molecules to B7-H3 and accordingly improve imaging contrast.

**Results:**

Two dimeric variants of anti-B7-H3 Affibody molecules were produced (designated Z_AC12*_-Z_AC12*_-GGGC and Z_AC12*_-Z_Taq_3_-GGGC). Both variants were labelled with Tc-99m (^99m^Tc) and demonstrated specific binding to B7-H3-expressing cells in vitro. [^99m^Tc]Tc-Z_AC12*_-Z_AC12*_-GGGC showed subnanomolar affinity (K_D1_=0.28 ± 0.10 nM, weight = 68%), which was 7.6-fold higher than for [^99m^Tc]Tc-Z_AC12*_-Z_Taq_3_-GGGC (K_D_=2.1 ± 0.9 nM). Head-to-head biodistribution of both dimeric variants of Affibody molecules compared with monomeric affinity matured SYNT-179 (all labelled with ^99m^Tc) in mice bearing B7-H3-expressing SKOV-3 xenografts demonstrates that both dimers have lower tumour uptake and lower tumour-to-organ ratios compared to the SYNT-179 Affibody molecule.

**Conclusion:**

The improved functional affinity by dimerization does not compensate the disadvantage of increased molecular size for imaging purposes.

**Supplementary Information:**

The online version contains supplementary material available at 10.1186/s41181-024-00261-3.

## Introduction

Non-invasive detection of overexpression of cell-surface proteins in malignant tumours can provide diagnostic information influencing patient stratification for specific therapies. Analysis of biopsy samples, a commonly used method to assess the expression level of molecular targets in solid tumours, is associated with several limitations caused by its invasiveness and heterogeneity of targets’ expression. Alternatively, radionuclide molecular imaging modalities such as single photon emission computed tomography (SPECT) and/or positron emission tomography (PET) could be used as non-invasive techniques to estimate expression levels of the cell-surface proteins enabling selection of targeted therapies and personalized anti-cancer treatment (Wu et al. 2023).

An emerging molecular target for cancer treatment is the immune checkpoint protein B7-H3 (Sun et al. 2002). This protein is involved in oncogenic signalling, tumour cell plasticity, and drug resistance. The protumourigenic activity of B7-H3 includes promotion of endothelial-to-mesenchymal transition, invasion, and angiogenesis (Zhou and Jin 2021). It should be noted that B7-H3 is overexpressed on differentiated malignant and cancer-initiating cells in many different cancer types (Roth et al. 2007; Zang et al. 2007, 2010; Crispen et al. 2008; Boorjian et al. 2008; Lemke et al. 2012; Ingebrigtsen et al. 2014; Sun et al. 2014; Bachawal et al. 2015; Inamura et al. 2018; Pulido and Nunes-Xavier 2023), while its expression level is low in most normal organs and tissues (Modak et al. 2001) making it an attractive molecular target for diagnostic and/or treatment applications.

Preclinical studies (Burvenich et al. 2018; Kasten et al. 2018) and phase I clinical trials (Modak et al. 2020; Kramer et al. 2022) using radiolabelled antibodies have demonstrated successful radionuclide targeting of B7-H3-expressing tumours. In spite of a specific tumour localisation and acceptable safety in patients, the bulkiness of such agents causes a relatively slow accumulation in tumours and clearance of activity from blood circulation. It means that several days are needed to obtain a reasonable imaging contrast. An alternative would be to reduce the size of the imaging agent, which is beneficial in term of better extravasation and faster clearance from blood and non-targeted sites (Garousi et al. 2020). The Affibody molecule is a small (6–7 kDa) engineered affinity protein based on a robust triple helical scaffold (Ståhl et al. 2017). Robustness, straightforward production and site-specific labelling are advantages of the use of Affibody molecules. The reduction of the size resulted in a high extravasation rate and fast clearance from blood providing high-contrast imaging a few hours after injection. In addition, small proteins are not accumulated in tumours due to the enhanced permeability and retention (EPR) effect (Wester and Kessler 2005), which increases specificity of imaging in comparison with imaging using monoclonal antibodies. These are essential advantages of Affibody molecules compared with monoclonal antibodies. Affibody molecules have been successfully evaluated as promising targeting agents for both preclinical (Orlova et al. 2006; Strand et al. 2014; Rosestedt et al. 2015; Garousi et al. 2016; Andersson et al. 2016; Oroujeni et al. 2018, 2021; Liu et al. 2022) and clinical (Sörensen et al. 2014, 2016; Alhuseinalkhudhur et al. 2023; Bragina et al. 2023) imaging of different molecular targets. Safety of this drug class has furthermore been demonstrated clinically with up to three years chronic patient dosing, suggesting viability for repeated administrations (Klint et al. 2023).

Proof-of principle of radionuclide visualization of expression levels of B7-H3 in vivo 2–4 h after injection was demonstrated using the [^99m^Tc]Tc-AC12-GGGC Affibody molecule (Oroujeni et al. 2022). It should be noted that the high sensitivity provided by the imaging agent is an essential precondition for successful translation of a radionuclide-based imaging agent into the clinic. The sensitivity depends on imaging contrast (tumour-to-organ ratios). These ratios are influenced by several characteristics of the imaging agent such as cellular retention of the label during cellular processing, tumour localization rate, clearance rate from normal organs, and biologic properties of the target. For development of new successful imaging agents, the focus should be on factors increasing the imaging contrast. This could be possible by both increasing tumour uptake and/or by decreasing uptake in non-targeted organs and tissues. affinity, i.e. a strength of binding to the desirable molecular target, is one of the crucial factors defining the efficacy of tumour targeting. Since the internalization of Affibody molecules is relatively slow (around 20–30% of cell-bound conjugate per day), the cellular retention of the radionuclide is strongly influenced by the binding strength of imaging agents to its target on a cancer cells surface (Wållberg and Orlova 2008; Tran et al. 2009). Cellular retention thus depends on the rate of dissociation of the tracer from target and on cellular processing of the tracer–target complex. Several studies have demonstrated that optimization of the binding strength of protein-based targeting probes to their tumour targets is critical for efficient tumour localization (Adams et al. 1998a; Adams et al. 1998b; Adams et al. 2001; Adams et al. 2006; Tolmachev et al. 2012). Affinity maturation can be used as an approach to improve affinity of Affibody molecules binding to different molecular targets, such as EGFR/HER1 (Friedman et al. 2008), HER2 (Orlova et al. 2006), HER3 (Malm et al. 2013) and PDGFRβ (Lindborg et al. 2011). Di- or multimerization is another approach for improving tumour-targeting properties of targeting probes, e.g. single-chain Fv fragments (Wu et al. 1996; Nielsen et al. 2000; Goel et al. 2001). This approach usually decreases the dissociation rate. This increases an apparent affinity of binding to a molecular target and might increase tumour localization of a number of targeting proteins (Wu et al. 1996; Adams et al. 1998b; Nielsen et al. 2000; Batra et al. 2002). However, there is a risk that an increase in size of the targeting molecule might outweigh the benefit of an increased affinity (Orlova et al. 2006; Tolmachev et al. 2009; Garousi et al. 2019).

The results of a recent study (Oroujeni et al. 2023) have shown that affinity maturation of anti-B7-H3 Affibody molecules resulted in an appreciably increased affinity compared to parental AC12 Affibody molecule. The use of these affinity matured Affibody molecules has shown an improved imaging contrast for imaging of B7-H3-expressing tumours in a murine model. However, once a promising targeting protein is found, further optimization of a tracer is often required.

**Fig. 1:**
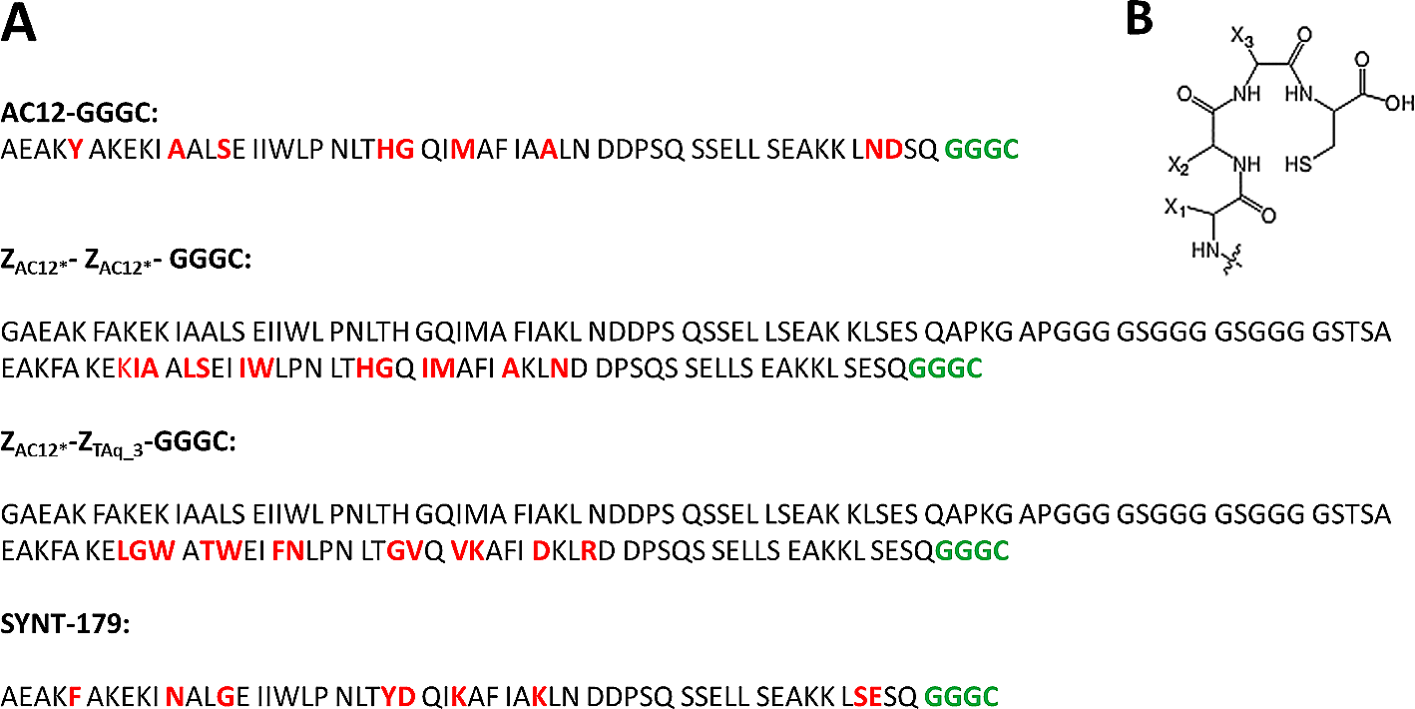
(**A**) Amino acid sequences of the evaluated Affibody molecules and (**B**) general structure of the N_3_S chelators formed by a C-terminal cysteine and three adjacent amino acids. X_1_, X_2_ and X_3_ are denoting the side chains of the amino acids (in this case X_1_,X_2_, X_3_ = hydrogen atom for glycine). The variable amino acids are marked in red and the chelator composition in green bold

The aim of this study was to find out, which approaches for increasing apparent affinity of B7-H3 Affibody molecules, dimerization or affinity maturation, would could improve the tumour-targeting properties of Affibody molecules for imaging of B7-H3-expressing tumours. In this study, according to previous studies (Altai et al. 2012; Oroujeni et al. 2021), peptide-based cysteine containing chelator –GGGC was incorporated at the C-terminus at Affibody molecules for labelling with ^99m^Tc. In this complex (Ahlgren et al. 2009; Altai et al. 2012), the amide nitrogens of amino acids from glycine and the thiol group of cysteine at peptide-based cysteine containing chelator form together a N_3_S chelator, providing a stable complex with ^99m^Tc (Fig. 1B). In order to compare different affinities at the same size, two dimeric variants of anti-B7-H3 Affibody molecules, Z_AC12*_-GGGC and Z_AC12*_-Z_Taq_3_-GGGC, were produced, were produced, characterized and labelled with ^99m^Tc as a commonly used radionuclide for SPECT imaging in clinics. The first variant, Z_AC12*_-Z_AC12*_-GGGC contained two units of the parental Z_AC12*_ Affibody molecule binding to B7-H3. To evaluate impact of size, the second variant contained only one B7-H3-binding unit and the second unit was Z_Taq_3_ Affibody molecule, which is a nonbinding Affibody. Z_Taq_ is an Affibody originally selected as a binder to Thermus aquaticus (Taq) DNA polymerase, which doesn’t interact with any mammalian proteins. Thus, Z_AC12*_-Z_Taq_3_-GGGC is a monovalent bi-Affibody. In vitro evaluation of new variants (testing specificity and affinity) was performed using B7-H3-expressing cell lines and compared with the affinity matured monomeric Affibody molecule SYNT-179 that was recently generated (Oroujeni et al. 2023).

## Materials and methods

### General

^99m^Tc as pertechnetate was obtained by elution of an Ultra TechneKow generator (Mallinckrodt, Petten, The Netherlands) with sterile 0.9% sodium chloride (Mallinckrodt, Petten, the Netherlands). For quantitative measurement of radioactivity distribution in instant thin-layer chromatography strips, a Cyclone Storage Phosphor system (Perkin-Elmer, Wellesley, MA, USA) was used. An automated gamma-spectrometer with a 3-inch NaI (TI) well detector (2480 Wizard, Wallac, Turku, Finland) was used to measure activity from cell and animal samples. A dose calibrator VDC-405 (Veenstra Instruments BV, Joure, The Netherlands) equipped with an ionization chamber was utilised for measuring the radioactivity for labelling and injection formulation.

B7-H3-expressing ovarian cancer SKOV-3 and breast cancer BT-474 cell lines, obtained from the American Type Culture Collection (ATCC), was used for in vitro cell studies. Ramos lymphoma cells (ATCC) were used to establish B7-H3-negative xenografts. Cells were cultured in RPMI medium (Flow Laboratories, Irvine, UK) supplemented with 10% of fetal bovine serum (20% of fetal bovine serum for BT-474), 2 mM of L-glutamine, 100 IU/mL of penicillin, and 100 mg/mL of streptomycin.

To determine significant differences (*p* < 0.05), in vitro studies and biodistribution data were analysed by unpaired 2-tailed t-test and ANOVA using GraphPad Prism (version 6 for Windows; GraphPad Software).

### Production, purification, and characterization of Novel Anti-B7-H3 Affibody Molecules

Genes encoding dimeric versions of a Z_AC−12_ were ordered from GeneArt (Thermo Fisher Scientific, MA, USA) as codon optimized and synthesized genes cloned in a custom plasmid to form fusion proteins with a His_6_ tag and a TEV protease cleavage site. After TEV protease treatment, resulting sequences were in the format G-Z_AC12*_- Z_AC12_-GGGC and G-Z_AC12*_- Z_Taq_3_-GGGC respectively.

Z_AC−12_ variants were expressed in autoinducing medium (Overnight Express TB, Novagen) inoculated with precultures of *E. coli* T7E2 clones carrying plasmids with sequence verified gene fragments of each B7-H3 binding Z_AC−12_ variant. Cells were disrupted, centrifuged and supernatants were used immediately for purification.

The lysates were purified using 1 mL His GraviTrap IMAC column (Cytiva). Contaminants were removed by washing with wash buffer and the Z_AC−12_ variants were subsequently eluted with elution buffer, with 1 mM dithiothreitol (DTT) added to all buffers. The Z_AC−12_ variants were buffer exchanged to 1 × DPBS with 1 mM DTT added and incubated over night at 4 °C with His-tagged TEV protease in a 20:1 molar ratio of Z_AC−12_ variant: TEV protease, and DTT was added to 2 mM final concentration. The incubated samples, supplemented with 20 mM imidazole, were applied on a 1 mL His GraviTrap IMAC column (Cytiva) equilibrated with binding buffer. TEV protease cleaved Z variants were collected in the flow through whereas His-tagged material bound to the IMAC resin. The non-tagged Z variants were further purified by reverse phase chromatography (RPC). and the buffer was exchanged to 1 × DPBS + 2 mM EDTA using PD-10 desalting columns (Cytiva).

RP-HPLC-MS analysis: Samples were analyzed using the Agilent 1290 Infinity UHPLC- system, equipped with single quadrupole and AP-ESI and the analytical column Zorbax 300SB-C8 RRHD (Agilent).

### Cell assays

The Affibody molecules SYNT-179, Z_AC12*_-Z_AC12*_-GGGC and Z_AC12*_-Z_Taq_3_-GGGC were tested in terms of binding to B7-H3 expressing SKOV-3 cells. The cells were placed in a V-bottom 96-well plate (0.2 × 10^6^ cells/well) and incubated at 4 °C for 1 h with Affibody molecules at decreasing concentrations from 555 nM to 28 pM. After 1× washing in PBS with 1% fetal bovine serum (FBS), binding of Affibody molecules was identified by an anti-Affibody polyclonal antibody (4 µg/mL) and incubated at 4 °C for 1 h followed by an Alexa488 conjugated goat-anti-rabbit IgG diluted 1:2000 and incubated at 4 °C for 1 h. After 2× washing in PBS with 1% FBS, fluorescence intensity was measured by a multimode plate reader (Enspire). Each sample was test-ed in quadruplicates. Binding curves were plotted and EC50 values were determined using software GraphPad Prism.

### Labelling with ^99m^Tc and in vitro stability of radioconjugates

Labelling of Affibody molecules with ^99m^Tc was performed using a lyophilized kit, as described earlier (Ahlgren et al. 2010). Briefly, a freeze-dried labelling kit containing 75 µg of tin (II) chloride dihydrate (Fluka Chemika, Buchs, Switzerland), 5 mg of gluconic acid sodium salt (Celsus Laboratories, Geel, Belgium), and 100 µg of ethylenediaminetetraacetic acid tetra sodium salt (EDTANa_4_) (Sigma-Aldrich, Munich, Germany) was prepared for labelling of Affibody molecules with ^99m^Tc. Radiolabelling of Affibody molecules was performed by adding the contents of the freeze-dried kit, dissolved in 120-µL degassed PBS, to 100 µg of the Affibody molecule. 80 µL (200–300 MBq) of ^99m^Tc-pertechnetate was added to the reaction mixture and degassed to protect the mixture from oxidation. The reaction vial was thoroughly vortexed and incubated at 90 °C for 1 h. Radiochemical yield of Affibody molecules was analysed using instant thin layer chromatography (ITLC-SG) (Agilent Technologies, Santa Clara, CA, USA) developed with PBS (Affibody: R_f_ = 0.0, other forms of ^99m^Tc: R_f_ = 1.0). The reduced hydrolysed technetium colloid (RHT) level in the labelling mixture was analysed using pyridine: acetic acid: water (10:6:3) as the mobile phase (^99m^Tc colloid: Rf = 0.0, other forms of ^99m^Tc and radio-labelled affibody molecule: Rf = 1.0). Since the radiochemical yield was more than 95% for all radioconjugates, no further purification was performed for in vitro and in vivo experiments.

To cross-validate radio-ITLC data further, reverse phase-HPLC conducted on an Elite LaChrom system (Hitachi, VWR, Darmstadt, Germany) consisting of an L-2130 pump, a UV detector (L-2400), and a radiation flow detector (Bioscan, Washington, DC, USA) coupled in series was used. Purity analysis of non-labelled and ^99m^Tc-labelled compounds was performed using an analytical column (Vydac RP C18 column, 300 Å; 3 × 150 mm; 5 μm). HPLC conditions were as follows: A = 10 mM TFA/H_2_O, B = 10 mM TFA/acetonitrile, UV-detection at 220 nm, gradient elution: 0–15 min at 5–70% B, 15–18 min at 70–95% B, 19–20 min at 5% B, and a flow rate was 1.0 mL/min.

To test in vitro stability, a fraction of a fresh radioconjugate (10 µL, 4 µg) was incubated with excess amount of PBS (40 µL) at 37 °C for 4 h. The test was performed in triplicates. To evaluate stability in blood serum, two samples of each radioconjugate (0.4 nmol) were mixed with the murine blood serum (100 µL) to mimic a concentration of the tracer in murine blood immediately after injection. Two control samples were mixed with the same amount of PBS. All samples were incubated at 37 ℃ for 4 h, and thereafter they were passed through NAP5 columns to separate a high molecular weight compounds (over 5 kDa) and low molecular weight compounds (less than 5 kDa). Activities in both high- and low-molecular weight fractions were measured to calculate percentage of ^99m^Tc associated with the high molecular weight fraction (i.e., bound to Affibody molecules).

### In vitro studies

Ovarian carcinoma SKOV-3 and breast carcinoma BT-474 cell lines as B7-H3-positive cells were used for in vitro studies. Ramos lymphoma cell line was used as a B7-H3-negative control for in vitro specificity and in vivo studies. B7-H3 expression levels were estimated to be 68000, 45000 and 250 receptors per cell for SKOV-3, BT-474 and Ramos, respectively (Oroujeni et al. 2022). Cells were seeded in cell-culture dishes (35 mm in diameter) with a density of 10^6^ cells/dish for in vitro study. A set of three dishes was used for in vitro binding specificity test.

To test in vitro binding specificity of each radioconjugate, cells in three control dishes were pre-saturated with 200-fold excess of a non-labelled Affibody molecule 15 min before addition of a radiolabelled conjugate. Cells in both blocked and nonblocked dishes were incubated with a radiolabelled conjugate (10 nM) in a humidified incubator (5% CO_2_, 37 °C) for 1 h. The medium was discarded, the cells were washed with cold serum-free medium before trypsin–EDTA solution (0.5 mL per dish) was added, and cells were additionally incubated for 10 min. Detached cells were diluted with 0.5 mL of complete medium, re-suspended, and transferred to fraction tubes. The radioactivity associated with cells was measured using an automated gamma counter and the cell-bound radioactivity was calculated.

Affinity of binding of each radiolabelled Affibody molecule to B7-H3 target was measured using a LigandTracer Yellow instrument (Ridgeview Instruments AB, Vänge, Sweden), as previously described (Björke and Andersson 2006). SKOV-3 cells (2 × 10^6^ cells/dish) were seeded on a local area of a cell culture dish (89 mm in diameter, NunclonTM, NUNC A/S, Roskilde, Denmark). The measurements were performed at room temperature to prevent internalization. Uptake curves were recorded for concentrations 1, 3 and 9 nM of ^99m^Tc-labelled conjugates. After incubation with labelled conjugate, the radioactive medium was replaced with fresh non-radioactive medium, and the dissociation curve was recorded for several hours. Analysis was performed in duplicates. The data were analysed using the Interaction Map software (Ridgeview Diagnostics AB, Uppsala, Sweden) to calculate the association rate, the dissociation rate, and the dissociation constant at equilibrium (K_D_). The basics of the InteractionMap analysis is described by Altschuh et al. (Altschuh et al. 2012).

### In vivo studies

Animal experiments were performed in accordance with the national legislation for work with laboratory animals. All animal studies were approved by the Ethics Committee for Animal Research in Uppsala (Sweden), following the national legislation on the protection of laboratory animals (permit 5.8.18–00473/2021, approved 26 February 2021). Four mice per data point were used in biodistribution experiments.

Biodistribution and targeting properties of ^99m^Tc-labelled Affibody conjugates were evaluated in BALB/C nu/nu mice bearing B7-H3-positive SKOV-3 xenografts. To establish xenografts, SKOV-3 cells (10^7^ cells/mouse) were subcutaneously injected on the right hind leg of female BALB/c nu/nu mice. For in vivo specificity, B7-H3-negative Ramos cells (5 × 10^6^ cells/mouse) were subcutaneously implanted on the left hind leg of female BALB/c nu/nu in mice. Biodistribution measurement was performed two weeks after cell implantation. Average animal weight was 17.9 ± 1.5 g. Average tumour weight was 0.27 ± 0.18 g and 0.8 ± 0.4 g for SKOV-3 and Ramos xenografts, respectively. The biodistribution was measured 4 h after injection in mice bearing SKOV-3 xenografts. Groups of 4 mice bearing tumour were injected with ^99m^Tc-labelled Affibody molecules (0.4 nmol, 7.8 µg, 60 kBq, 100 µL in PBS) into the tail vein. To test B7-H3-specific accumulation, one group of animals bearing B7-H3-negative Ramos xenografts was injected with the same peptide and activity doses for each conjugate and the biodistribution was measured 4 h after injection. After 4 h, mice were euthanized by overdosing of anaesthetic solution (20 µL of solution per gram of body weight: ketamine, 10 mg/mL; Xylazine, 1 mg/mL). This was followed by a heart puncture, and blood samples were collected. Organs and tissue samples were collected and weighed. Organ radioactivity was measured using a gamma spectrometer along with three standards of injected activity and empty syringes for each animal. Uptake values for organs were calculated as the percentage of injected dose per gram tissue (%ID/g). For comparison, one group of mice was injected with monomeric Affibody molecule SYNT-179 labelled with ^99m^Tc (0.4 nmol, 3 µg, 60 kBq, 100 µL in PBS) into the tail and biodistribution was measured 4 h after injection. To show in vivo binding specificity, two groups of mice bearing SKOV-3 xenografts were intraperitoneally injected with 600 µg of Affibody molecules 40 min before injection of the best binders, [^99m^Tc]Tc-Z_AC12*−_Z_AC12*−_GGGC and [^99m^Tc]Tc-SYNT-179. The biodistribution was measured 4 h after injection as described above.

To confirm biodistribution results, a small animal SPECT/CT imaging was performed. One SKOV-3 bearing mouse was intravenously injected with 14 MBq/0.4 nmol of ^99m^Tc-labelled Affibody molecule. To confirm in vivo specificity, one mouse bearing Ramos xenograft was intravenously injected with the same mass and activity of ^99m^Tc-labelled Affibody molecule. The mice were imaged 4 h after injection using a nanoSPECT/CT scanner (Mediso Medical Imaging Systems, Budapest, Hungary). The mice were euthanized by CO_2_ asphyxiation immediately before being placed in the camera. The computed tomography (CT) acquisition was carried out at the following parameters: energy peak of 50 kV, 670 µA, 480 projections, and 2.29-min acquisition time. SPECT acquisition was performed at the following parameters: ^99m^Tc energy peak of 140 keV, window width of 20%, matrix of 256 × 256, and acquisition time of 1 h. CT images were reconstructed in real-time using Nucline 2.03 Software (Mediso Medical Imaging Systems, Budapest, Hungary). SPECT raw data were reconstructed using TeraTomo™ 3D SPECT reconstruction technology.

## Results

### Production, purification, and characterization of novel Anti-B7-H3 affibody molecule

To evaluate targeting properties of Affibody molecule labelled with ^99m^Tc, the peptide-based cysteine containing –GGGC chelator was incorporated at the C-terminus of Affibody molecules. Figure 1B shows the general structure of the N_3_S chelator formed by a C-terminal cysteine and three adjacent amino acids (triglycine-cysteine, – GGGC, chelator). The sequences of Affibody molecules, which were evaluated in this study, as well as the sequence of the parental Z_AC−12_-GGGC Affibody molecules are shown in Fig. 1A.

According to LC-MS analysis, purities were 96 and 98% for Z_AC12*_-Z_AC12*_-GGGC and Z_AC12*_-Z_Taq_3_-GGGC, respectively. The correct masses were confirmed for Z_AC12*_-Z_AC12*_-GGGC and Z_AC12*_-Z_Taq_3_-GGGC by LC-MS.

**Fig. 2:**
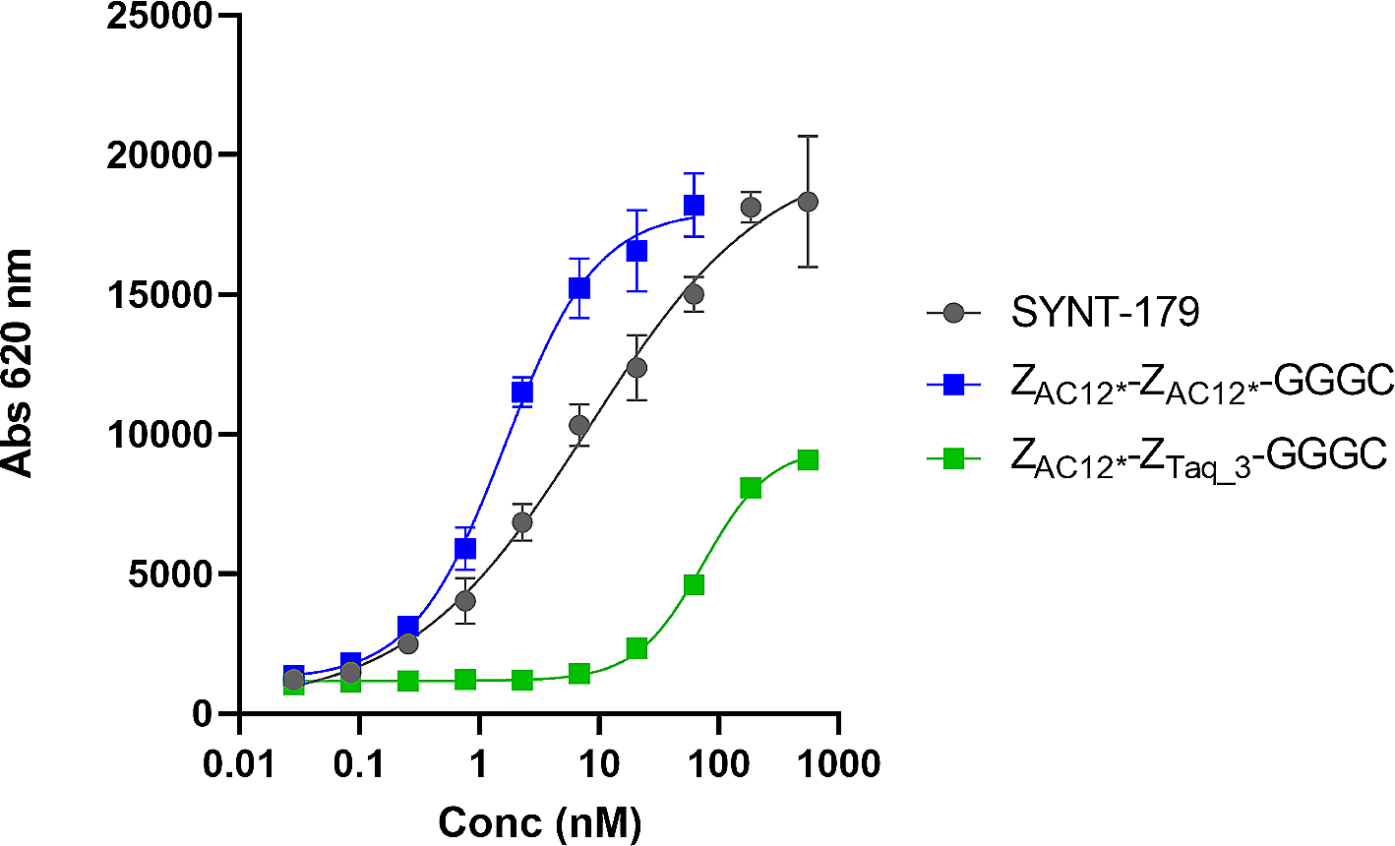
Determination of the EC_50_ of dimeric variants and SYNT-179 binding to B7-H3-expressing SKOV-3 cells

The results of EC_50_ determination are presented in Fig. 2. The data suggest that SYNT-179 had stronger binding to SKOV-3 cells (EC_50_ = 8.1 nM) than heterodimeric Z_AC12*_-Z_Taq_3_-GGGC (EC_50_ = 73.6 nM). However, the binding of homodimeric Z_AC12*_-Z_AC12*_-GGGC was stronger (EC_50_ = 1.5 nM) than the binding of SYNT-179.

### Labelling with ^99m^Tc and in vitro stability of radioconjugates

Labelling of dimeric anti-B7-H3 Affibody molecules with ^99m^Tc was successfully performed providing the radiochemical yield exceeding 95% and radiocolloid concentration below 5% (Table 1). The specific activity of ^99m^Tc-labelled conjugates was 3 MBq/µg. Because of the high radiochemical purity of the labelled constructs, no additional purification was performed for in vitro and in vivo studies. ^99m^Tc-labelled Affibody molecules were stable during incubation at 37 °C for 4 h in the presence of excess of PBS (Table 1).

After 4-h incubation of in murine serum at 37 °C, 94.3 ± 3.6, 96.9 ± 0.3 and 91.3 ± 0.5% of technetium was associated with the high molecular weight fraction (molecular weight over 5 kDa) for [^99m^Tc]Tc-Z_AC12*_-Z_AC12*_-GGGC, [^99m^Tc]Tc-Z_AC12*_-Z_Taq_3_-GGGC and [^99m^Tc]Tc-SYNT-179, respectively. In control samples, which were diluted in PBS to the same concentration and incubated in the same condition, 96.4 ± 0.9, 98.8 ± 1.7 and 92.1 ± 0.7% of technetium was associated with the high molecular fraction for [^99m^Tc]Tc-Z_AC12*_-Z_AC12*_-GGGC, [^99m^Tc]Tc-Z_AC12*_-Z_Taq_3_-GGGC and [^99m^Tc]Tc-SYNT-179, respectively.

**Table 1:** Labelling of Affibody molecules with ^99m^Tc and in vitro stability

	Radiochemical yield, %	Stability in PBS, 37 °C, 4 h
Z_AC12*_-Z_AC12*_-GGGC	97.4 ± 1.1	94.5 ± 0.2
Z_AC12*_-Z_Taq_3_-GGGC	97.6 ± 1.5	94.3 ± 1.5
SYNT-179	99.3 ± 0.4	95 ± 1

According to radiochromatogram, both ^99m^Tc-labelled conjugates were eluted as single peaks with the retention time of between 12 and 13 min (Fig. S1) with a good match to the retention time of non-labelled standards (ultraviolet detector).

### In vitro studies

**Fig. 3:**
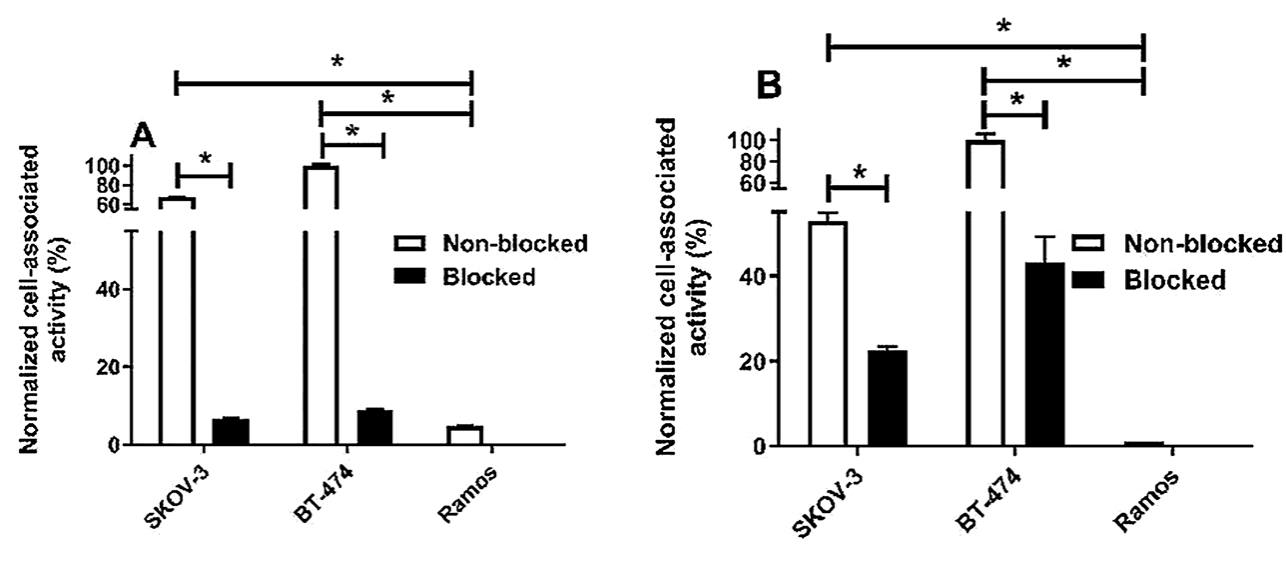
In vitro binding specificity of (**A**) [^99m^Tc]Tc-Z_AC12*_-Z_AC12*_-GGGC and (**B**) [^99m^Tc]Tc-Z_AC12*_-Z_Taq_3_-GGGC by SKOV-3 and BT-474 cells. For the pre-saturation of B7-H3, a 200-fold molar excess of the same non-labelled Z_AC12*_-Z_AC12*_-GGGC and Z_AC12*_-Z_Taq_3_-GGGC Affibody molecules was added to the dishes before adding the corresponding radiolabelled conjugate, respectively. Data are normalized to the average value of cell-associated radioactivity for non-blocked value for BT474. Asterisk (*) marks significant (*p* < 0.05) difference. The data are presented as an average value from three samples ± SD

In vitro binding specificity of [^99m^Tc]Tc-Z_AC12*_-Z_AC12*_-GGGC and [^99m^Tc]Tc-Z_AC12*_-Z_Taq_3_-GGGC to B7-H3-expressing cells was measured by a saturation experiment. The binding was significantly (*p* < 0.005) decreased when the cells were pre-saturated with the excess amount of non-labelled anti-B7-H3 Affibody molecule (Fig. 3). The binding of both variants was significantly (*p* < 0.005) lower on Ramos as a negative-B7-H3 cell line compared with B7-H3-positive cell lines (SKOV-3 and BT-474) confirming the binding level is proportional to the expression level of the target.

**Fig. 4:**
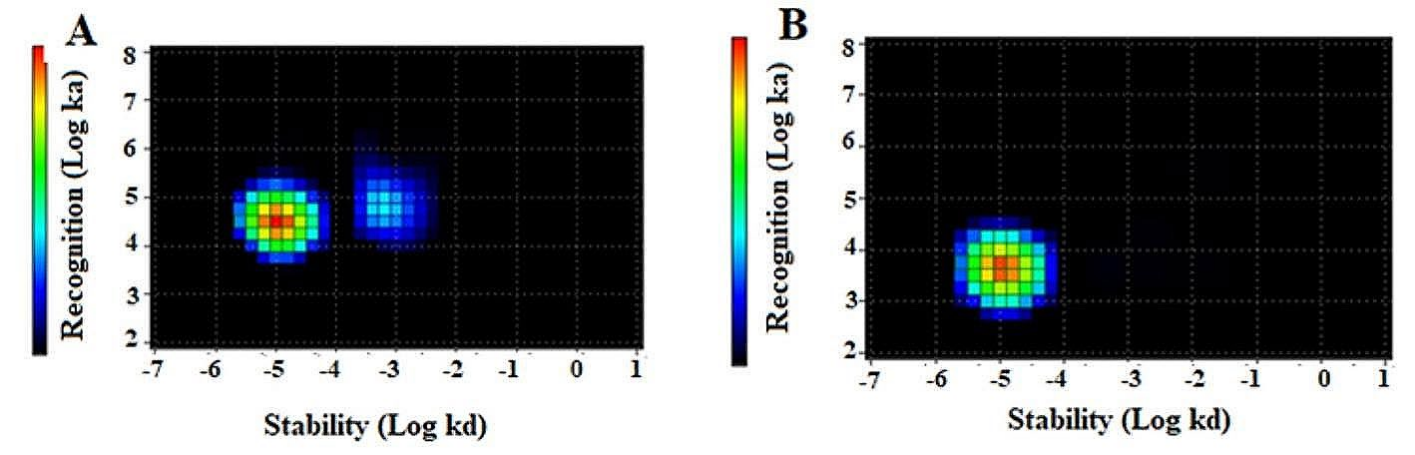
Interaction Map of (**A**) [^99m^Tc]Tc-Z_AC12*_-Z_AC12*_-GGGC and (**B**) [^99m^Tc]Tc-Z_AC12*_-Z_Taq_3_-GGGC on SKOV-3 cells. Input data were obtained from LigandTracer measurement of cell-bound activity during association of labelled conjugate to- and dissociation from SKOV-3 cells. Binding was measured at three different concentrations, 1, 3 and 9 nM. Measurements were performed in duplicates

The results of binding kinetics of [^99m^Tc]Tc-Z_AC12*_-Z_AC12*_-GGGC and [^99m^Tc]Tc-Z_AC12*_-Z_Taq_3_-GGGC to B7-H3-expressing SKOV-3 cells measured in real-time are presented in Fig. 4; Table 2. According to the assay results, the best fit of the binding of [^99m^Tc]Tc-Z_AC12*_-Z_AC12*_-GGGC to SKOV-3 cell line was achieved using a 1:2 model, suggesting that there were two types of interactions with B7-H3. [^99m^Tc]Tc-Z_AC12*_-Z_AC12*_-GGGC showed a major affinity in subnanomolar range (K_D1_= 0.28 ± 0.10 nM, % weight = 68%). The best fit of the binding of [^99m^Tc]Tc-Z_AC12*_-Z_Taq_3_-GGGC to SKOV-3 cells was achieved using a 1:1 model and it was in nanomolar range (K_D_=2.1 ± 0.9 nM).

**Table 2:** Apparent equilibrium dissociation (K_D_) constants for the interaction between ^99m^Tc-labelled Affibody molecules and B7-H3-expressing SKOV-3 cells determined using an Interaction Map analysis of the LigandTracer sensorgrams.* Data are taken from (Oroujeni et al. 2022, 2023)

	K_D1_ (nM)	Weight_1_, %	K_D2_ (nM)	Weight_2_, %
[^99m^Tc]Tc-Z_AC12-_GGGC*	1.9 ± 0.8	23	68.8 ± 7.4	67
[^99m^Tc]Tc-Z_AC12*_-Z_AC12*_-GGGC	0.28 ± 0.10	68	15.2 ± 6.3	32
[^99m^Tc]Tc-Z_AC12*_-Z_Taq_3_-GGGC	2.1 ± 0.9	100	-	-
[^99m^Tc]Tc-SYNT-179*	0.028 ± 0.001	12	8.2 ± 0.5	88

### In vivo studies

**Fig. 5:**
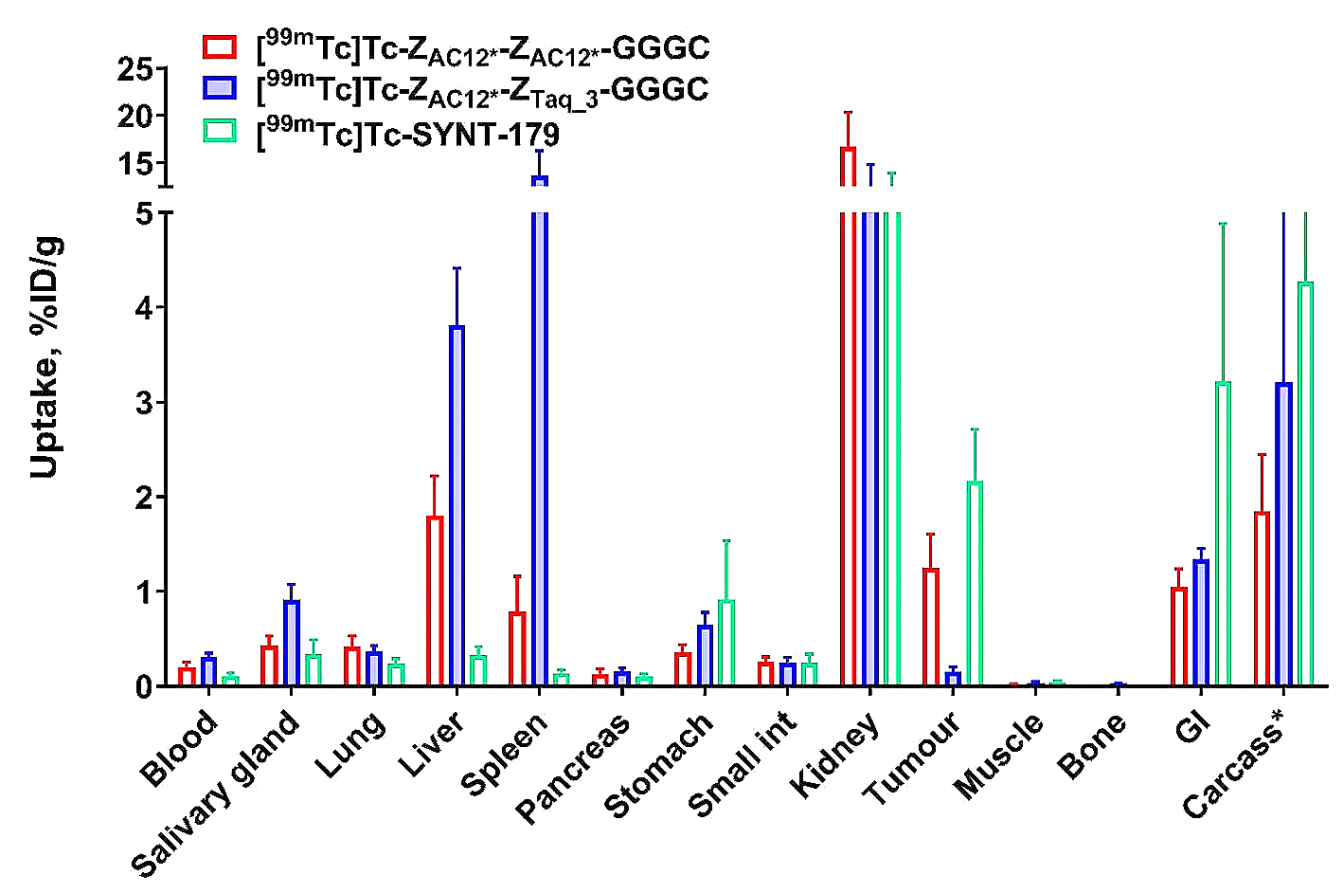
Comparative biodistribution of ^99m^Tc-labelled Affibody molecules in BALB/C nu/nu mice bearing SKOV-3 xenografts 4 h after injection. Data are expressed as the percentage of administered activity (injected probe) per gram of tissue (% ID/g). The data are presented as the average (*n* = 4) and SD

The results of a head-to-head comparison of biodistribution of two dimeric variants of B7-H3 Affibody molecules (Z_AC12*_-Z_AC12*_-GGGC and Z_AC12*_-Z_Taq_3_-GGGC) and the best monomeric variant (SYNT-179) labelled with ^99m^Tc at 4 h after injection in tumour-bearing mice are presented in Fig. 5 and supplemental Table 1. Biodistribution data demonstrated significantly (*p* < 0.05) lower blood concentration for Z_AC12*_-Z_AC12*_-GGGC than for Z_AC12*_-Z_Taq_3_-GGGC. There was no significant difference (*p* > 0.05) in blood concentration for Z_AC12*_-Z_AC12*_-GGGC and SYNT-179. The tumour uptake of [^99m^Tc]Tc-Z_AC12*_-Z_AC12*_-GGGC (1.25 ± 0.36%ID/g) and [^99m^Tc]Tc-SYNT-179 (2.17 ± 0.54%ID/g) was significantly (*p* < 0.05) higher than that for [^99m^Tc]Tc- Z_AC12*_-Z_Taq_3_-GGGC (0.15 ± 0.05%ID/g). However, there was no significant difference between tumour uptake of [^99m^Tc]Tc-Z_AC12*_-Z_AC12*_-GGGC and [^99m^Tc]Tc-SYNT-179 (*p* > 0.05). The hepatic uptake was significantly (*p* < 0.05) lower for [^99m^Tc]Tc-Z_AC12*_-Z_AC12*_-GGGC (1.80 ± 0.42%ID/g) than for [^99m^Tc]Tc-Z_AC12*_-Z_Taq_3_-GGGC (3.81 ± 0.60%ID/g). Still, [^99m^Tc]Tc-SYNT-179 showed significantly (*p* < 0.05) lower hepatic uptake (0.33 ± 0.09%ID/g) in comparison to both dimeric variants. The renal uptake was at the same level for all radioconjugates (16.74 ± 3.61%ID/g, 12.04 ± 2.82%ID/g and 11.87 ± 2.08 for [^99m^Tc]Tc-Z_AC12*_-Z_AC12*_-GGGC, [^99m^Tc]Tc-Z_AC12*_-Z_Taq_3_-GGGC and [^99m^Tc]Tc-SYNT-179, respectively). Dimeric variants had a tendency for lower accumulation in the intestines with content, although their uptake in small intestine wall did not differ significantly.

**Fig. 6:**
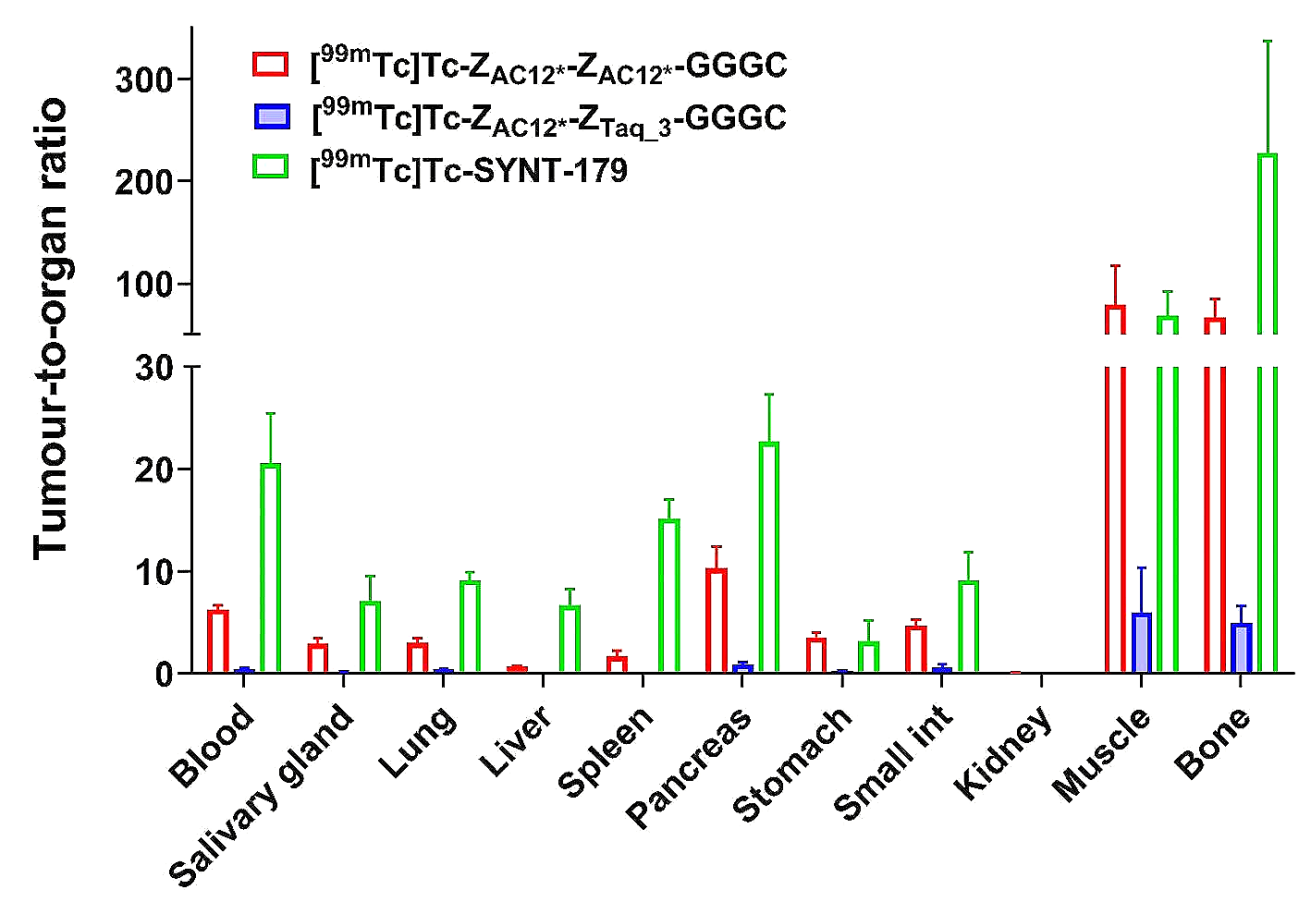
Tumour-to-organ ratios of ^99m^Tc-labelled Affibody molecules in BALB/C nu/nu mice bearing SKOV-3 xenografts 4 h after injection. The data are presented as the average (n = 4) and SD

The data concerning tumour-to-organ ratios of ^99m^Tc-labelled Affibody molecules in tumour-bearing mice 4 h after injection are presented in Fig. 6 and supplemental Table 2. Faster blood clearance and higher tumour uptake resulted in significantly (*p* < 0.05) higher tumour-to-blood ratio for [^99m^Tc]Tc-Z_AC12*_-Z_AC12*_-GGGC (6.25 ± 0.42) compared to [^99m^Tc]Tc-Z_AC12*_-Z_Taq_3_-GGGC (0.47 ± 0.10) at 4 h after injection. However, [^99m^Tc]Tc-SYNT-179 showed significantly (*p* < 0.05) higher tumour-to-blood ratio (20.55 ± 4.95) than both dimeric variants. Higher tumour uptake and less uptake in almost all organs and tissues resulted in significantly (*p* < 0.05) higher tumour-to-organ ratios for [^99m^Tc]Tc-Z_AC12*_-Z_AC12*_-GGGC than for [^99m^Tc]Tc-Z_AC12*_-Z_Taq_3_-GGGC. For example, we observed significantly (*p* < 0.05) higher tumour-to-liver (0.69 ± 0.06 vs. 0.04 ± 0.01), tumour-to-muscle (79.12 ± 38.12 vs. 6.00 ± 4.33), tumour-to-bone (66.77 ± 18.25 vs. 4.99 ± 1.63) ratios for [^99m^Tc]Tc-Z_AC12*_-Z_AC12*_-GGGC compared to those for [^99m^Tc]Tc-Z_AC12*_-Z_Taq_3_-GGGC. However, almost 2-fold higher tumour uptake of [^99m^Tc]Tc-SYNT-179 and less uptake in almost all organs and tissues resulted in significantly (*p* < 0.05) higher tumour-to-blood (20.55 ± 4.95), tumour-to-lung (9.14 ± 0.83), tumour-to-liver (6.68 ± 1.64), tumour-to-spleen (15.15 ± 1.85), tumour-to-pancreas (22.70 ± 4.65) ratios than those for [^99m^Tc]Tc-Z_AC12*_-Z_AC12*_-GGGC (6.25 ± 0.42, 3.01 ± 0.42, 0.69 ± 0.06, 1.70 ± 0.51 and 10.29 ± 2.11, respectively).

**Fig. 7:**
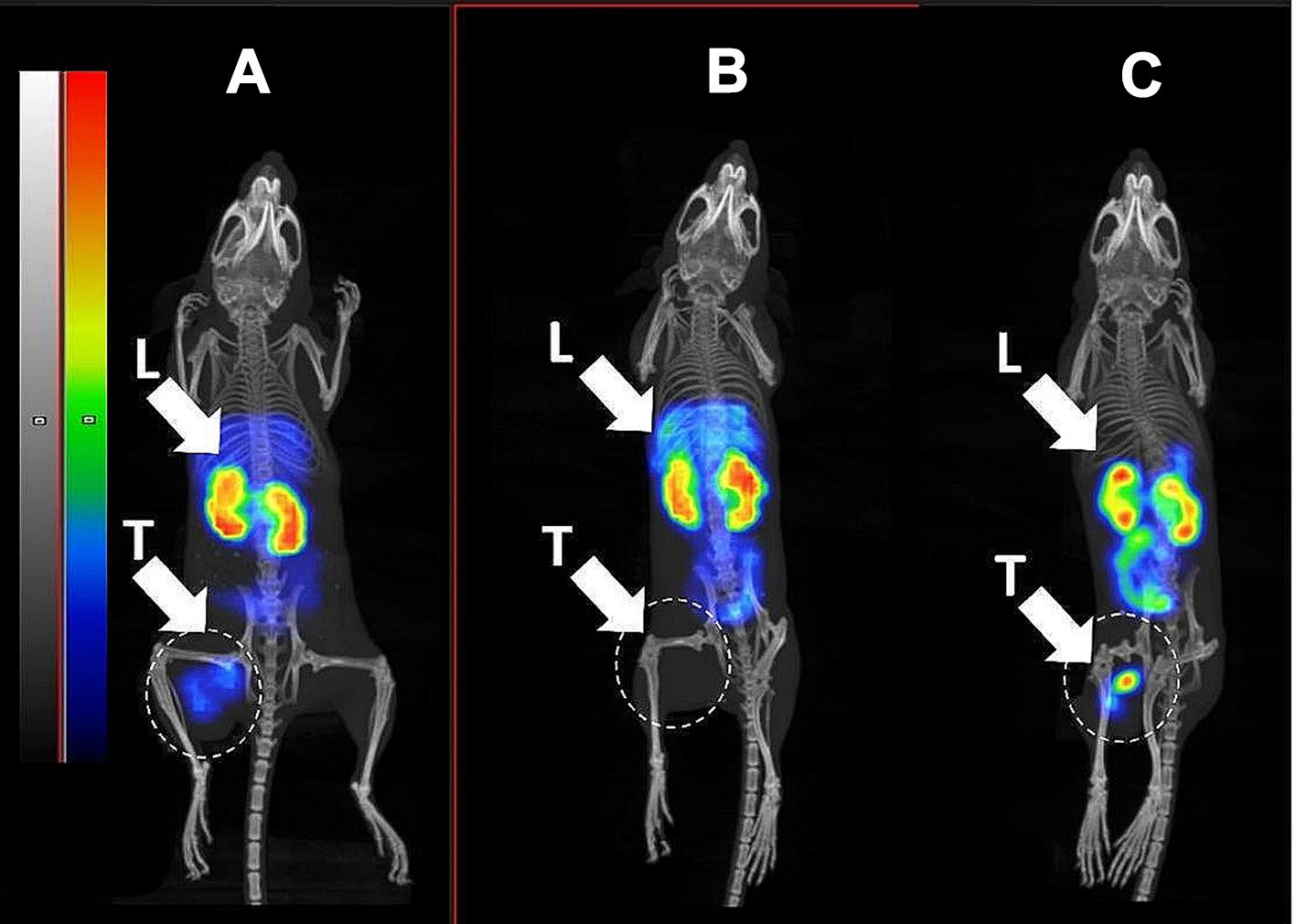
Imaging of (**A**) [^99m^Tc]Tc-Z_AC12*_-Z_AC12*_-GGGC (**B**) [^99m^Tc]Tc-Z_AC12*_-Z_Taq_3_-GGGC and (**C**) [^99m^Tc]Tc-SYNT-179 in BALB/C nu/nu mice bearing B7-H3-positive SKOV-3 xenografts 4 h after injection. Linear relative scale (arbitrary units normalized to a maximum count rate) is provided for each image. Scales were adjusted to show red pixels in xenografts. Arrows point at tumour (T) and liver (L)

Results of nanoSPECT/CT imaging (Fig. 7) of all three radioconjugates in BALB/C nu/nu mice bearing B7-H3-positive SKOV-3 xenografts 4 h after injection confirmed ex vivo biodistribution measurements data. A pronouncedly less accumulation of activity in liver and higher accumulation in tumour for [^99m^Tc]Tc-SYNT-179 compared to [^99m^Tc]Tc-Z_AC12*_-Z_AC12*_-GGGC and [^99m^Tc]Tc-Z_AC12*_-Z_Taq_3_-GGGC were observed. [^99m^Tc]Tc-Z_AC12*_-Z_Taq_3_-GGGC had the highest accumulation in liver.

**Fig. 8:**
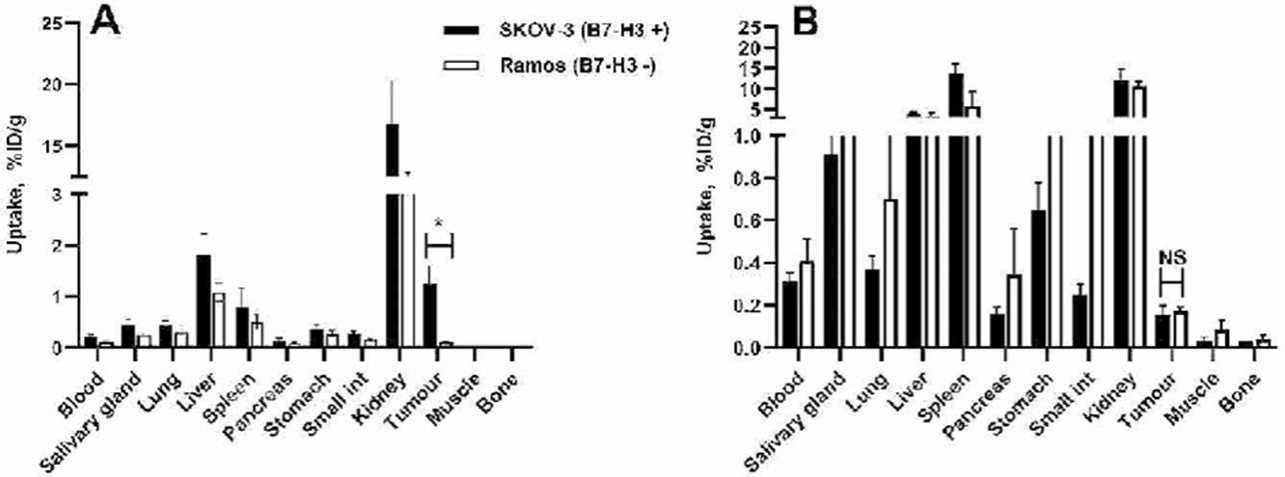
Uptake of (**A**) [^99m^Tc]Tc-Z_AC12*_-Z_AC12*_-GGGC and (**B**) [^99m^Tc]Tc-Z_AC12*−_Z_Taq_3_-GGGC on SKOV-3 (B7-H3-positive) and Ramos (B7-H3-negative) xenografts at 4 h after injection. Data are expressed as %ID/g and are averages from four mice ± SD. Asterisk (*) marks significant (*p* < 0.05) difference

To test in vivo specificity of [^99m^Tc]Tc-Z_AC12*_-Z_AC12*_-GGGC and [^99m^Tc]Tc-Z_AC12*_-Z_Taq_3_-GGGC, the tumour accumulation in B7-H3-positive SKOV-3 xenografts and in B7-H3-negative Ramos xenografts was compared (Fig. 8 and Supplemental Table 3). The uptake of [^99m^Tc]Tc-Z_AC12*_-Z_AC12*_-GGGC in SKOV-3 xenografts was significantly (*p* < 0.005) higher than in Ramos. This shows that the tumour accumulation was dependent on expression of B7-H3. No significant difference in tumour uptake between B7-H3-positive SKOV-3 and B7-H3-negative Ramos xenografts for [^99m^Tc]Tc-Z_AC12*_-Z_Taq_3_-GGGC was observed (Fig. 8B and Supplemental Table 3).

**Fig. 9:**
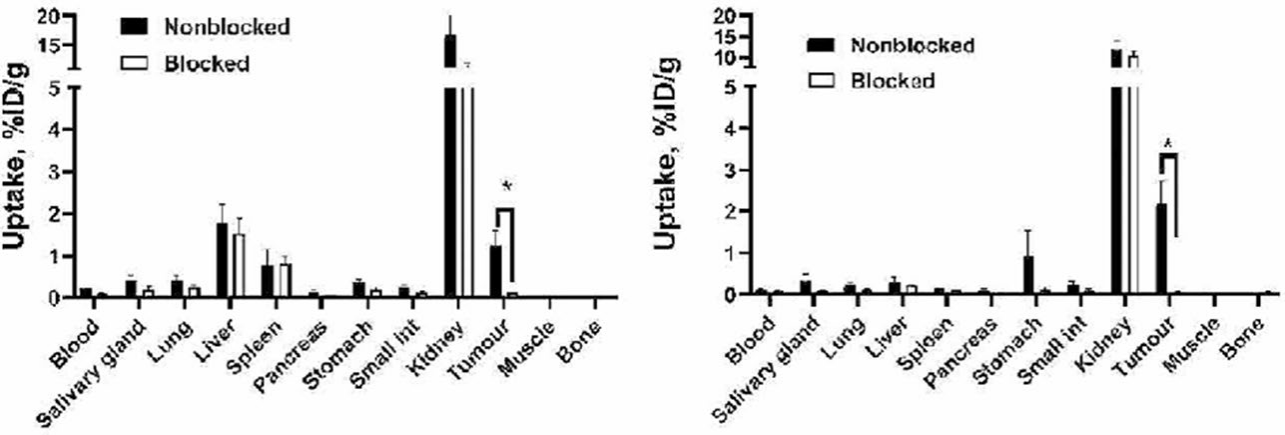
Uptake of (**A**) [^99m^Tc]Tc-Z_AC12*_-Z_AC12*_-GGGC and (**B**) [^99m^Tc]SYNT-179 on SKOV-3 (B7-H3-positive) xenografts at 4 h after injection. B7-H3 was blocked with pre-injection of 600 µg of Affibody molecule 40 min before injection of the radioconjugate. Data are expressed as %ID/g and are averages from four mice ± SD. Asterisk (*) marks significant (*p* < 0.05) difference

The results of blocking of B7-H3 using preinjcetion of Affibody molecule before injection of the [^99m^Tc]Tc-Z_AC12*_-Z_AC12*_-GGGC and [^99m^Tc]Tc-SYNT-179 in mice bearing SKOV-3 as B7-H3-positive xenografts (Fig. 9) showed that the tumour uptake for both radioconjugates reduced when B7-H3 is blocked by injection of Affibody molecule before injection the radiolabelled conjugate, confirming B7-H3-specific binding in vivo.

**Fig. 10:**
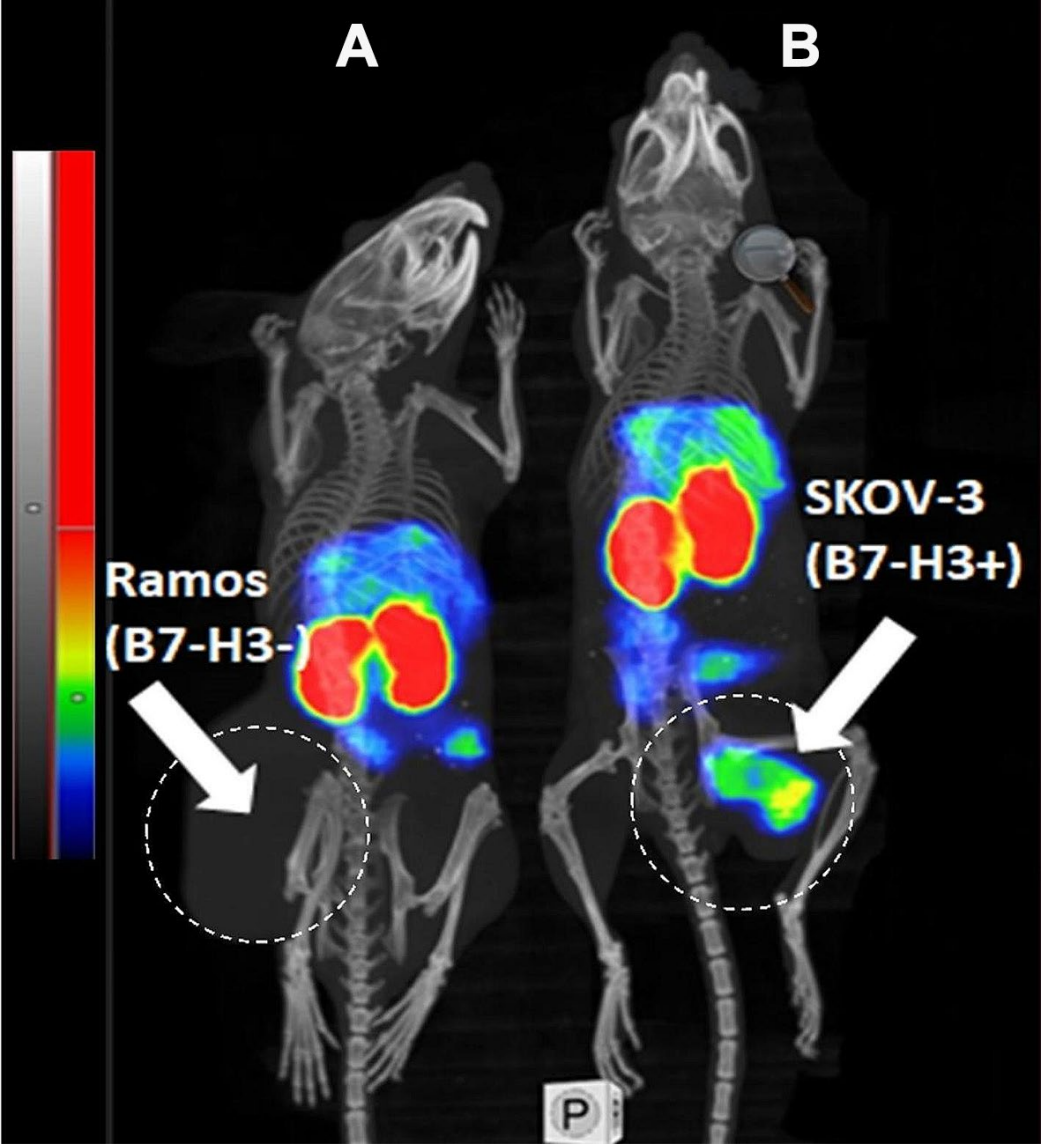
Imaging of [^99m^Tc]Tc-Z_AC12*_-Z_AC12*_-GGGC in BALB/C nu/nu mice bearing (**A**) B7-H3-negative Ramos xenografts and (**B**) B7-H3-positive SKOV3 xenografts 4 h after injection. 7.8 µg of labelled Affibody molecule (14 MBq, 100 µL in PBS) was injected into the tail vein. Arrows point at tumours (T). The animals were imaged simultaneously. Linear relative scale (arbitrary units normalized to a maximum count rate) was adjusted to visualize B7-H3-positive SKOV-3 xenograft

The results of ex vivo measurements were also confirmed by nanoSPECT/CT imaging (Fig. 10). Activity uptake of [^99m^Tc]Tc-Z_AC12*_-Z_AC12*_-GGGC in B7-H3-negative Ramos xenografts (Fig. 10A), was appreciably lower than in the SKOV-3 xenograft (Fig. 10B).

## Discussion

The small size and engineered excellent binding specificity of scaffold proteins make them promising targeting vectors for radionuclide molecular imaging (Garousi et al. 2020; Tolmachev et al. 2022). The favourable imaging properties of Affibody molecules were confirmed by development of the B7-H3 imaging probe [^99m^Tc]Tc-Z_AC12−_GGGC (Oroujeni et al. 2022), which in a mouse model provided better tumour-to-blood ratio (11.0 ± 0.5) already 4 h after injection than the conventional antibody-based tracer ^89^Zr-DS-5573a (Burvenich et al. 2018) provided 7 days after injection (5.03 ± 0.73). Still, scaffold proteins are much less studied for imaging than antibodies, and influence of different parameters on their properties should be further elucidated.

Affinity is an essential feature of tracers (Eckelman et al. 2006) and how affinity is optimized is important. To improve the imaging properties of the Affibody-molecule-mediated imaging of B7-H3, an affinity maturation was performed. The affinity maturation resulted in improved monomeric affinity and a two-fold increase of tumour-to-blood ratio as compared to the parental Z_AC12_ construct (Oroujeni et al. 2023). Dimeric formatting of a targeting molecule is another strategy to increase the apparent affinity and may be easier and faster to accomplish. In this study, we compared the effects of affinity maturation and a dimeric format on imaging properties of B7-H3-targeting Affibody molecules. As control we used the parental Z_AC12_ molecule as monomer binding but in dimeric format with a Taq polymerase binding Affibody molecule to create a construct of the same size as the high affinity dimer but with parental affinity. The two monomeric constructs, Z_AC12_ and SYNT-179, have been compared previously (Oroujeni et al. 2023).

Despite a seeming simplicity, the dimeric formatting might be associated with some pitfalls. For example, selection of inappropriate linkers might hamper a proper folding of the target-binding domains and reduce the affinity of a dimer (Garousi et al. 2019). However, the use of a trimeric-(G_3_S)_3_- linker in this study permitted to obtain a binding-competent dimer. Another risk is that one or both of the binding domains become sterically hindered from interacting with the epitope on the target molecule. In this case, [^99m^Tc]Tc-Z_AC12*_-Z_Taq_3_-GGGC preserved the affinity of the strongest interaction of parental [^99m^Tc]Tc-Z_AC12_-GGGC (1.9 nM) with positive B7-H3 cells (Table 2). Moreover, the abundance of the strongest interaction increased from 23 to 100% for [^99m^Tc]Tc-Z_AC12*_-Z_Taq_3_-GGGC. The reason for this unexpected positive effect is not quite clear, but it is thinkable that the non-binding ZTaq_3 domain could help stabilize the binding interaction. On the other hand, the detection efficiency of secondary reagents to the heterodimeric Z-scaffold may have been impacted, which can be suggested from cell binding experiments in Fig. 2 where the absolute signal seems to be lower than for the other constructs. With a successful design of the architecture of the construct, the homodimeric formatting had a clear positive effect on the binding strength of Z_AC12*_-Z_AC12*_-GGGC as its half-inhibition concentration (EC_50_) decreased nearly 50-fold compared with the Z_Taq_-containing variant. The EC_50_ of Z_AC12*_-Z_AC12*_-GGGC was also 5.4-fold better than for the monomeric affinity matured SYNT-179. The EC_50_ data were concordant with the measurements of kinetics of binding to the living cells (Table 2), where the apparent dissociation constant for the strongest interaction of [^99m^Tc]Tc- Z_AC12*_-Z_AC12*_-GGGC was 7-fold lower compared with the strongest interaction of monomer [^99m^Tc]Tc-Z_AC12_-GGGC.

The affinity maturation enabled to obtain lower K_D_ values for the strongest interaction of [^99m^Tc]Tc-SYNT-179 compared with both parental [^99m^Tc]Tc-Z_AC12−_GGGC and dimeric [^99m^Tc]Tc-Z_AC12*_-Z_AC12*_-GGGC, but this high-affinity interaction was relatively less abundant (K_D1_=0.028 ± 0.001nM, %weight_1_ = 12). The most abundant interaction of [^99m^Tc]Tc-SYNT-179 (K_D2_=8.2 ± 0.5 nM, %weight_2_ = 88) was 8-fold stronger that the most abundant interaction of [^99m^Tc]Tc-Z_AC12_-GGGC (K_D2_=68.8 ± 7.4, %weight_2_ = 67). Still, a direct interpretation of kinetic measurement might be complicated, because the human B7-H3 has two identical domains and, accordingly, two potential epitopes. It could also not be excluded that binding to one epitope causes conformational changes, which modify affinity to another epitope. Overall, the EC_50_ might be a better measure for comparison of binding strength, and the results of EC_50_-measurements were more in favour of the dimeric format.

Still, in vivo data are crucial for selection of the optimal format of an imaging probe. The biodistribution data from this study (Figs. 5 and 6) were in a good agreement (with overlapping of errors) with similar data obtained in earlier experiments. For example, the tumour uptake of [^99m^Tc]Tc-SYNT-179 in this study was 2.17 ± 0.54%ID/g, while Oroujeni et al. (Oroujeni et al. 2023) reported the uptake 1.54 ± 0.19%ID/g in SKOV-3 xenografts at the same time point. The tumour-to-blood ratios were 20.6 ± 5.0 in this study and 25.7 ± 2.5 in the earlier investigation.

The uptake of [^99m^Tc]Tc-Z_AC12*_-Z_AC12*_-GGGC in B7-H3-positive SKOV-3 xenografts was 12-fold higher than in B7-H3-negative Ramos xenografts (Fig. 8A), which clearly demonstrated that the tumour uptake in vivo was B7-H3-mediated. These data were confirmed by imaging results (Fig. 10A and B). Despite stronger binding of [^99m^Tc]Tc-Z_AC12*_-Z_AC12*_-GGGC in vitro, no significant difference in tumour uptake for [^99m^Tc]Tc-Z_AC12*_-Z_AC12*_-GGGC and the affinity maturated monomer [^99m^Tc]Tc-SYNT-179 was observed (*p* > 0.05 in ANOVA test with Bonferroni’s correction for multiple comparisons). Moreover, there was a tendency to lower tumour uptake of the dimeric variant [^99m^Tc]Tc-Z_AC12*_-Z_AC12*_-GGGC. Furthermore, the tumour uptake of [^99m^Tc]Tc-Z_AC12*_-Z_AC12*_-GGGC (1.25 ± 0.3%ID/g) was not better than the tumour uptake of historical data for [^99m^Tc]Tc-Z_AC12−_GGGC (1.04 ± 0.08%ID/g at the same time point) reported earlier (Oroujeni et al. 2022) despite higher binding strength of the [^99m^Tc]Tc-Z_AC12*_-Z_AC12*_-GGGC. Some insight into this phenomenon might be obtained by comparison with targeting properties of [^99m^Tc]Tc-Z_AC12*_-Z_Taq_3_-GGGC. The affinity of this construct was (at least) not worse than the affinity of [^99m^Tc]Tc-Z_AC12_-GGGC, but its tumour uptake was lower (0.15 ± 0.05%ID/g) than the uptake of [^99m^Tc]Tc-Z_AC12_-GGGC (Oroujeni et al. 2022). This suggests that the small size of an Affibody-based construct is critical for maximizing a tumour uptake. A possible explanation can be that the extravasation rate is essential for scaffold proteins, which are cleared rapidly from circulation. This rapid clearance results in a disappearance of the concentration gradient between blood and tumour. It is quite likely, that the increase of the size of a dimer slows down the extravasation during the critical time when the tracer’s concentration in blood is still high (Schmidt and Wittrup 2009). This decrease cannot be compensated by improvement of the affinityImportantly, previous studies using Affibody molecules for targeting HER2 have also demonstrated that keeping the size small might be more important than gaining functional affinity by dimerization (Orlova et al. 2006; Tolmachev et al. 2009). The influence of dimerization on targeting properties of a series of HER2-targeting ADAPT molecules has similarly showed that despite higher affinity, dimers had lower tumour uptake and lower tumour-to-organ ratios compared to the monomer (Garousi et al. 2019). If the size penalty observed in this study is of general value, it suggests that even relatively small affinity molecules of 14 kDa size e.g. nanobodies and DARPins, may have similar extravasation penalty and therefore lower tumour uptake.

## Conclusion

To conclude, increase in affinity by dimeric formatting in order to improve targeting properties of an anti-B7-H3 Affibody molecule was not efficient. It appears that increase of affinity should be performed by affinity maturation in order to keep the molecular format as small as possible. The affinity matured SYNT-179 Affibody molecule is hence a more favourable B7-H3-specific Affibody molecule than the dimeric variants tested herein for imaging of B7-H3-expressing tumours in vivo.

### Electronic supplementary material

Below is the link to the electronic supplementary material.

Supplementary Material 1

Supplementary Material 2

## Data Availability

Data is contained within the article or supplementary material.
