# Supplementary material for: Comparison of approaches for increasing affinity of affibody molecules for imaging of B7-H3: dimerization and affinity maturation
Source: EJNMMI Radiopharm Chem. 2024 Apr 16;9:30. doi: 10.1186/s41181-024-00261-3 (PMC11021382; doi:10.1186/s41181-024-00261-3)
Supplement: Supplementary file 1 — Supplementary Material 1 [file 41181_2024_261_MOESM1_ESM.docx]

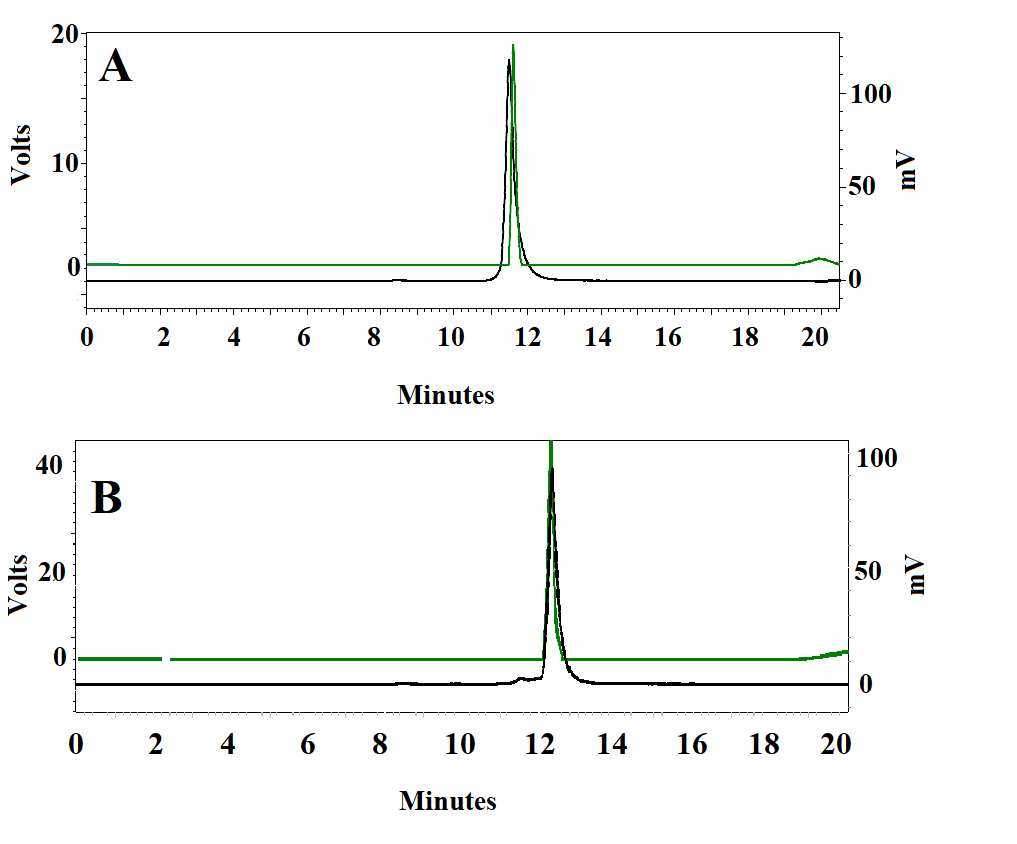


**Supplemental Fig. 1.** Characterization of anti-B7-H3 Affibody molecules. Reversed-phase HPLC chromatogram of non-labelled (green) and radiochromatogram (black) of ^99m^Tc-labelled of (A) Z_AC12*_-Z_AC12*_-GGGC and (B) Z_AC12*_-Z_Taq_3_-GGGC Affibody molecules. The retention times (Rt) are expressed in minutes.

**Supplemental Table 1.** Comparative biodistribution of ^99m^Tc-labelled Affibody molecules in BALB/C nu/nu mice bearing SKOV-3 xenografts 4 h after injection. Data are expressed as the percentage of administered activity (injected probe) per gram of tissue (% ID/g). The data are presented as the average (n = 4) and SD. Error bars might not be seen because they are smaller than the symbols.

| **Site** | **[^99m^Tc]Tc-Z_AC12*_-Z_AC12*_-GGGC** | **[^99m^Tc]Tc-Z_AC12*_-Z_Taq_3_-GGGC** | **[^99m^Tc]Tc-SYNT-179** |
| --- | --- | --- | --- |
| Blood | 0.20±0.05^a^ | 0.31±0.04^a,c^ | 0.11±0.03^c^ |
| Salivary glands | 0.43±0.10 | 0.91±0.16^c^ | 0.34±0.15^c^ |
| Lung | 0.42±0.11 | 0.37±0.06 | 0.24±0.05 |
| Liver | 1.80±0.42^a,b^ | 3.81±0.60^a,c^ | 0.33±0.09^b,c^ |
| Spleen | 0.79±0.37^a^ | 13.68±2.6^a,c^ | 0.14±0.03^c^ |
| Pancreas | 0.13±0.05 | 0.16±0.03 | 0.10±0.03 |
| Stomach | 0.36±0.08 | 0.65±0.13 | 0.92±0.61 |
| Small intestine | 0.26±0.05 | 0.25±0.05 | 0.25±0.09 |
| Kidney | 16.74±3.61 | 12.04±2.82 | 11.87±2.08 |
| Tumour | 1.25±0.36^a^ | 0.15±0.05^a,c^ | 2.17±0.54^c^ |
| Muscle | 0.02±0.01 | 0.03±0.02 | 0.04±0.02 |
| Bone | 0.019±0.00^a,b^ | 0.030±0.003^a,c^ | 0.010±0.002^b,c^ |
| Intestines with content* | 1.05±0.19 | 1.34±0.11 | 3.22±1.66 |
| Carcass* | 1.85 ± 0.60 | 3.21 ± 2.12 | 4.27 ± 1.95 |

^a^ Significant difference (*p* < 0.05) between [^99m^Tc]Tc-Z_AC12*_-Z_AC12*_-GGGC and [^99m^Tc]Tc-Z_AC12*_-Z_Taq_3_-GGGC; ^b^ significant difference (*p* < 0.05) between [^99m^Tc]Tc-Z_AC12*_-Z_AC12*_-GGGC and [^99m^Tc]Tc- SYNT-179; ^c^ significant difference (*p* < 0.05) between [^99m^Tc]Tc-Z_AC12*_-Z_Taq_3_-GGGC and [^99m^Tc]Tc-SYNT-179. ANOVA test (Bonferroni's multiple comparisons test) was performed to test significant (*p* < 0.05) difference. * Data for intestines with content and carcass are presented as % of injected dose per whole sample, excluding a small segment of small intestine.

**Supplemental Table 2**. Tumour-to-organ ratios of ^99m^Tc-labelled Affibody molecules in BALB/C nu/nu mice bearing SKOV-3 xenografts 4 h after injection. The data are presented as the average (n = 4) and SD.

| **Site** | **[^99m^Tc]Tc-Z_AC12*_-Z_AC12*_-GGGC** | **[^99m^Tc]Tc-Z_AC12*_-Z_Taq_3_-GGGC** | **[^99m^Tc]Tc-SYNT-179** |
| --- | --- | --- | --- |
| Blood | 6.25±0.42^a,b^ | 0.47±0.10^a,c^ | 20.55±4.95^b,c^ |
| Salivary glands | 2.92±0.49^a^ | 0.16±0.04^a,c^ | 7.12±2.39^c^ |
| Lung | 3.01±0.42^a,b^ | 0.40±0.10^a,c^ | 9.14±0.83^b,c^ |
| Liver | 0.69±0.06^a,b^ | 0.04±0.01^a,c^ | 6.68±1.64^b,c^ |
| Spleen | 1.70±0.51^a,b^ | 0.011±0.003^a,c^ | 15.15±1.85^b,c^ |
| Pancreas | 10.29±2.11^a,b^ | 0.93±0.17^a,c^ | 22.70±4.65^b,c^ |
| Stomach | 3.52±0.46^a^ | 0.23±0.07^a^ | 3.17±2.01 |
| Small intestine | 4.69±0.60^a^ | 0.63±0.26^a,c^ | 9.15±2.67^c^ |
| Kidney | 0.08±0.02 ^a,b^ | 0.013±0.004^a,c^ | 0.18±0.03^b,c^ |
| Muscle | 79.12±38.12^a^ | 6.00±4.33^a,c^ | 68.77±23.61^c^ |
| Bone | 66.77±18.25^a^ | 4.99±1.63^a^ | 227.84±109.54 |

^a^ Significant difference (*p* < 0.05) between [^99m^Tc]Tc-Z_AC12*_-Z_AC12*_-GGGC and [^99m^Tc]Tc-Z_AC12*_-Z_Taq_3_-GGGC; ^b^ significant difference (*p* < 0.05) between [^99m^Tc]Tc-Z_AC12*_-Z_AC12*_-GGGC and [^99m^Tc]Tc- SYNT-179; ^c^ significant difference (*p* < 0.05) between [^99m^Tc]Tc-Z_AC12*_-Z_Taq_3_-GGGC and [^99m^Tc]Tc-SYNT-179. ANOVA test (Bonferroni's multiple comparisons test) was performed to test significant (*p* < 0.05) difference.

**Supplemental Table 3**. Biodistribution of ^99m^Tc-labelled Affibody molecules in BALB/C nu/nu mice bearing SKOV-3 (B7-H3 positive) and Ramos (B7-H3 negative) xenografts 4 h after injection. Data are expressed as the percentage of administered activity (injected probe) per gram of tissue (% ID/g). The data are presented as the average (n = 4) and SD.

|  | **[^99m^Tc]Tc-Z_AC12*_-Z_AC12*_-GGGC** | | **[^99m^Tc]Tc-Z_AC12*_-Z_Taq_3_-GGGC** | |
| --- | --- | --- | --- | --- |
| **Site** | **SKOV-3** | **Ramos** | **SKOV-3** | **Ramos** |
| Blood | 0.20±0.05 | 0.10±0.02 | 0.31±0.04 | 0.41±0.1 |
| Salivary glands | 0.43±0.10 | 0.24±0.02 | 0.91±0.16 | 1.43±0.60 |
| Lung | 0.42±0.11 | 0.30±0.05 | 0.37±0.06 | 0.70±0.40 |
| Liver | 1.80±0.42 | 1.08±0.17 | 3.81±0.60 | 3.40±0.83 |
| Spleen | 0.79±0.37 | 0.50±0.15 | 13.68±2.6 | 6.10±3.23 |
| Pancreas | 0.13±0.05 | 0.08±0.02 | 0.16±0.03 | 0.34±0.22 |
| Stomach | 0.36±0.08 | 0.27±0.05 | 0.65±0.13 | 1.58±0.71 |
| Small intestine | 0.26±0.05 | 0.15±0.01 | 0.25±0.05 | 1.08±0.22 |
| Kidney | 16.74±3.61 | 12.03±0.87 | 12.04±2.82 | 10.69±1.16 |
| Tumour | 1.25±0.36^*^ | 0.10±0.0^*^ | 0.15±0.05 | 0.17±0.02 |
| Muscle | 0.02±0.01 | 0.015±0.004 | 0.03±0.02 | 0.08±0.05 |
| Bone | 0.019±0.00 | 0.015±0.003 | 0.030±0.003 | 0.04±0.02 |

* Significant difference (*p* < 0.05) between SKOV-3 (B7-H3 positive) and Ramos (B7-H3 negative) xenografts; T-test was performed to test significant difference. No significant difference in tumour uptake between SKOV-3 and Ramos for [^99m^Tc]Tc-Z_AC12*_-Z_Taq_3_-GGGC was observed.
