# Supplementary material for: Comparison of approaches for increasing affinity of affibody molecules for imaging of B7-H3: dimerization and affinity maturation
Source: EJNMMI Radiopharm Chem. 2024 Apr 16;9:30. doi: 10.1186/s41181-024-00261-3 (PMC11021382; doi:10.1186/s41181-024-00261-3)
Supplement: Supplementary file 2 — Supplementary Material 2 [file 41181_2024_261_MOESM2_ESM.docx]

Reviewer #1: Manuscript entitled "Comparison of Approaches for Increasing Affinity of Affibody Molecules for Imaging of B7-H3: Dimerization and Affinity Maturation" describes development of two dimeric variants of anti-B7-H3 Affibody molecules labeled with 99mTc and comparison of biological evaluation data with monomeric affinity matured 99mTc labeled SYNT-179 molecule. Although similar article by same group discussed development of affinity matured variants of affibody towards B7-H3, present study compares the dimeric variants with affinity matured monomer, mainly influence of higher molecular weight on pharmakokinetics of such radiotracers is evaluated.

Overall very systematic study featuring robust data from animal models. Conducting additional studies to validate the stability in human serum and assess the in vivo stability of the radiotracers will further enhance the strength of the results.

**Answer. Thank you for pointing out this important aspect! To address this comment, we have evaluated stability in human serum and the following text is included to the Material and methods and Results sections of the revised manuscript: “**To evaluate stability in blood serum, two samples of each radioconjugate (0.4 nmol) were mixed with the murine blood serum (100 µL) to mimic a concentration of the tracer in murine blood immediately after injection. Two control samples were mixed with the same amount of PBS. All samples were incubated at 37 ℃ for 4 h, and thereafter they were passed through NAP5 columns to separate a high molecular weight compounds (over 5 kDa) and low molecular weight compounds (less than 5 kDa). Activities in both high- and low-molecular weight fractions were measured to calculate percentage of ^99m^Tc associated with the high molecular weight fraction (i.e., bound to Affibody molecules) **“**.

**“**After 4-h incubation of in murine serum at 37 °C, 94.3±3.6, 96.9±0.3 and 91.3±0.5 % of technetium was associated with the high molecular weight fraction (molecular weight over 5 kDa) for [^99m^Tc]Tc-Z_AC12*_-Z_AC12*_-GGGC, [^99m^Tc]Tc-Z_AC12*_-Z_Taq_3_-GGGC and [^99m^Tc]Tc-SYNT-179, respectively. In control samples, which were diluted in PBS to the same concentration and incubated in the same condition, 96.4±0.9, 98.8±1.7 and 92.1±0.7 % of technetium was associated with the high molecular fraction for [^99m^Tc]Tc-Z_AC12*_-Z_AC12*_-GGGC, [^99m^Tc]Tc-Z_AC12*_-Z_Taq_3_-GGGC and [^99m^Tc]Tc-SYNT-179, respectively. **“**

**Concerning in vivo stability. The use of the GGGC peptide-based chelator in combination with ^99m^Tc or ^188^Re results in the formation of a non-residualizing label (see e.g. Wållberg H et al, J Nucl Med. 2011 Mar;52(3):461-9; Mitran B et al. Amino Acids. 2015 Feb;47(2):303-15; Oroujeni M et al. Amino Acids. 2018 Aug;50(8):981-994; Oroujeni M et al. Int J Mol Sci. 2021 Mar 9;22(5):2770; Altai M et al. . Eur J Med Chem. 2014 Nov 24;87:519-28; Liu Y et al. Pharmaceutics. 2022 May 20;14(5):1092.). Affibody molecules are reabsorbed from primary urine and internalized in the renal proximal tubuli. Typically, 70-80% of the injected tracer is reabsorbed. The use of the non-residualizing 99mTc-GGGC label results in the rapid release of radiometabolites from kidney and reduces the renal uptake of labelled affibody molecules. However, a part of these radiometabolites entry the blood. These metabolites would obscure the results of the in vivo stability test. Therefore, we did not perform such a test in this study.**

Wållberg H, Orlova A, Altai M, Hosseinimehr SJ, Widström C, Malmberg J, Ståhl S, Tolmachev V. Molecular design and optimization of 99mTc-labeled recombinant affibody molecules improves their biodistribution and imaging properties. J Nucl Med. 2011 Mar;52(3):461-9;

Mitran B, Altai M, Hofström C, Honarvar H, Sandström M, Orlova A, Tolmachev V, Gräslund T. Evaluation of 99mTc-Z IGF1R:4551-GGGC affibody molecule, a new probe for imaging of insulin-like growth factor type 1 receptor expression. Amino Acids. 2015 Feb;47(2):303-15;

Oroujeni M, Andersson KG, Steinhardt X, Altai M, Orlova A, Mitran B, Vorobyeva A, Garousi J, Tolmachev V, Löfblom J. Influence of composition of cysteine-containing peptide-based chelators on biodistribution of 99mTc-labeled anti-EGFR affibody molecules. Amino Acids. 2018 Aug;50(8):981-994;

Oroujeni M, Rinne SS, Vorobyeva A, Loftenius A, Feldwisch J, Jonasson P, Chernov V, Orlova A, Frejd FY, Tolmachev V. Preclinical Evaluation of 99mTc-ZHER2:41071, a Second-Generation Affibody-Based HER2-Visualizing Imaging Probe with a Low Renal Uptake. Int J Mol Sci. 2021 Mar 9;22(5):2770;

Altai M, Honarvar H, Wållberg H, Strand J, Varasteh Z, Rosestedt M, Orlova A, Dunås F, Sandström M, Löfblom J, Tolmachev V, Ståhl S. Selection of an optimal cysteine-containing peptide-based chelator for labeling of affibody molecules with (188)Re. Eur J Med Chem. 2014 Nov 24;87:519-28;

Liu Y, Vorobyeva A, Orlova A, Konijnenberg MW, Xu T, Bragina O, Loftenius A, Rosander E, Frejd FY, Tolmachev V. Experimental Therapy of HER2-Expressing Xenografts Using the Second-Generation HER2-Targeting Affibody Molecule 188Re-ZHER2:41071. Pharmaceutics. 2022 May 20;14(5):1092

Minor grammatical mistakes and formatting errors, especially in the reference section, require attention. pdf file with comments is attached.

**Answer. Thank you for pointing out this! The changes suggested by the reviewer have been implemented into the revised version of the manuscript.**

Although competition studies with cold affibody confirms specificity, Studies in B7-H3 negative cell line would have further strengthened the results.

**Answer. Thank you for pointing out this. The binding of both variants was performed on Ramos as a negative cell line and the following texts were added to the ‘’Materials and Methods’’ and ‘’Result’’ sections of the revised version of the manuscript:**

**Materials and Methods, 2.4. In vitro:** ‘’Ovarian carcinoma SKOV-3 and breast carcinoma BT-474 cell lines as B7-H3-positive cells were used for in vitro studies. Ramos lymphoma cell line was used as a B7-H3-negative control for in vitro specificity and in vivo studies. ‘’

**In Results, 3.3. In vitro studies:** ‘’The binding of both variants was significantly (p < 0.005) lower on Ramos as a negative-B7-H3 cell line compared with B7-H3-positive cell lines (SKOV-3 and BT-474) confirming the binding level is proportional to the expression level of the target.’’

Fig. 3 In vitro binding specificity of A) [99mTc]Tc-ZAC12*-ZAC12*-GGGC and B) [99mTc]Tc-ZAC12*-ZTaq_3-GGGC by SKOV-3 and BT-474 cells. For the pre-saturation of B7-H3, a 200-fold molar excess of the same non-labelled ZAC12*-ZAC12*-GGGC and ZAC12*-ZTaq_3-GGGC Affibody molecules was added to the dishes before adding the corresponding radiolabelled conjugate, respectively. Data are normalized to the average value of cell-associated radioactivity for non-blocked value for BT474. Asterisk (*) marks significant (p<0.05) difference .The data are presented as an average value from three samples ± SD.

**Concerning comments added to the PDF file.**

**A comment in the Introduction**

Soluble B7-H3 (sB7-H3), which is cleaved from the surface by a matrix metallopeptidase (MMP) or produced through the alternative splicing of the intron, has also been detected in human sera" for future clinical translation of radiotracer, presence of such soluble B7-H3 might show higher blood uptake in patients.....

Chen W, Liu P, Wang Y, Nie W, Li Z, Xu W, et al. Characterization of a Soluble B7-H3 (sB7-H3) Spliced From the Intron and Analysis of sB7-H3 in the Sera of Patients With Hepatocellular Carcinoma. PloS One (2013) 8:e76965. doi: 10.1371/journal.pone.0076965

13. Zhang G, Hou J, Shi J, Yu G, Lu B, Zhang X. Soluble CD276 (B7-H3) is Released From Monocytes, Dendritic Cells and Activated T Cells and is Detectable in Normal Human Serum. Immunology (2008) 123:538–46. doi: 10.1111/j.1365-2567.2007.02723.x

**Answer. We understand the reviewer’s concern. Indeed a soluble or “shed” target might be an issue for the radionuclide targeting since it can intercept the targeting probe in the blood circulation. However, it has been demonstrated that increasing the injected mass of a radiolabeled anti-mesothelin antibody enables saturation of the shed mesothelin in the blood reducing the blood concentration and the hepatic uptake and increasing tumor uptake (see Lee et al 2015). This has to be definitely considered during the planning of clinical translation of our tracers. However, we feel that this topic is not quite relevant to this paper and should be discussed separately. It has to be noted that the presence of a shed antigen.**

Lee JH, Kim H, Yao Z, Lee SJ, Szajek LP, Grasso L, Pastan I, Paik CH. Tumor and organ uptake of (64)Cu-labeled MORAb-009 (amatuximab), an anti-mesothelin antibody, by PET imaging and biodistribution studies. Nucl Med Biol. 2015 Nov;42(11):880-6.

**A comment in the Results**

The in vitro stability of three conjugates in PBS after 4 h incubation at 37 ℃ was added to Table 1.

**A comment in the Discussion**

**“**Dimers could be retained for longer duration at the target site and might be better utilized for therapeutic application. **“**

**Answer. We agree that the apparent dissociation rate of dimers is typically lower by an order of magnitude compared with the rate of the corresponding monomers. Of course, this might be utilized for a targeted therapy. However, it is premature to discuss the use of application of the current format of B7-H3-binding Affibody molecules for therapy. The renal uptake is much higher than the uptake in kidneys. Therefore, we focus on the imaging applications. For this purpose, dimer are worse than monomers because of an impaired delivery.**

Reviewer #2: The authors describe a study comparing monovalent and bivalent affibody targeting B3H7.

I do not understand the concept of the study and therefore I also assume that the conclusions in the paper can be hardly drawn with these studies. Why is the bivalent affibody for B3H7 ZAC12*-ZAC12*-GGGC compared to SYNT-179 which is an affinity matured affibody with a 10-fold higher affinity than ZAC12*-ZAC12*-GGGC (Kd-results)? I also would like to point out that affinity measurement of bivalent compounds is very difficult and may strongly depend on concentration and cell surface distribution of the target! Therefore, the affinity of bivalent compounds depends on the system.

**Answer. Thank you for pointing out this interesting aspect! The reviewer stated that “I also would like to point out that affinity measurement of bivalent compounds is very difficult and may strongly depend on concentration and cell surface distribution of the target! Therefore, the affinity of bivalent compounds depend on the system.“ The issue is even more complicated, as there are up to four epitopes on B7-H3 protein. It might be that K_D_ measurements would not reflect all aspects. EC50 shown stronger binding of ZAC12*-ZAC12*-GGGC compared to SYNT-179.**

It does not make sense to change the affibody in the different groups. The logical project would be to compare the same affibody as a monovalent di-affibody (ZAC12*-ZTaq_3-GGGC) and a bivalent di-affibody (ZAC12*-ZAC12*-GGGC) and as a control ZAC12*-GGGC. Or the same compounds with SYNT-179 (which would be for me the most logic approach).

**Answer. Thank you for pointing out this important aspect! It might be that we have not formulated the goal clearly. Abstract. “**In this study, we tested the hypothesis that a dimeric format may be an alternative option to increase the apparent affinity of Affibody molecules to B7-H3 and accordingly improve imaging contrast**.” We have rewritten the last paras of the Introduction section to stress that our idea was to understand what is better, dimerization or affinity maturation. Dimerization is quick but increases size, maturation is lengthy, but keeps size small. In fact, we wanted to see which approach is better for targeting in vivo.** **To address this comment, the following text is included to the Introduction section of the revised manuscript: “**The aim of this study was to find out, which approaches for increasing apparent affinity of B7-H3 Affibody molecules, dimerization or affinity maturation, would could improve the tumour-targeting properties of Affibody molecules for imaging of B7-H3-expressing tumours. ”

Is the bivalent SYNT-179 better than the monovalent! Therefore, from my point of view it is not allowed to draw this conclusion:
"The improved functional affinity by dimerization does not compensate the disadvantage of increased molecular size for imaging purposes."
"It appears that increase of affinity should be performed by affinity maturation in order to keep the molecular format as small as possible." This conclusion is not valid and cannot concluded out of these data. This are two different approaches to improve radiolabeled compounds and can be used not only parallel as is done here but also serial.

**Answer. Thank you for pointing out this! One can do this serial, but then a long affinity maturation would be required. We wanted to see if the maturation can be avoided completely.**

**The comparison between AC12 monomer and the affinity matured SYNT-179 has been done and was recently described (Oroujeni 2023). It was found that affinity maturation improved tumor uptake, although moderately (2.15% ID/g versus 1.04%ID/g at 4 hours). Given the dimeric nature of B7-H3, it could make sense to investigate whether a dimerization of the construct would yield better results than the affinity maturation for the monomer. In this context, the natural comparison molecule is the affinity matured SYNT-179. It is then also logical to try to understand the size impact of dimerization by adding as control the monovalent di-Affibody. Indeed, in in vitro cell binding experiments, the bivalent construct (ZAC12*-ZAC12*-GGGC) seemed superior as compared to monomer di-Affibody (ZAC12*-ZTaq_3-GGGC) or monomer matured single Affibody (SYNT-179). The KD on cells was also almost identical for the monomer AC12 and monomer di-Affibody (ZAC12*-ZTaq_3-GGGC), supporting use of monomer di-Affibody (ZAC12*-ZTaq_3-GGGC) as control. To our surprise, the improvement in affinity did not pay off in vivo. To summarize, monomeric ZAC12 and SYNT-179 have already been compared in vivo (Oroujeni 2023) and it hence makes sense to study two novel affinity improved constructs side by side, monomer versus dimer and similar affinities. With the limitation in use of animals and given the body of already existing data on the monomer ZAC12, we felt that it was relevant to use SYNT-179 as monomer control in this study.**

**We have clarified the text in the manuscript as follows, Introduction:’’** In order to compare different affinities at the same size, two dimeric variants of anti-B7-H3 Affibody molecules, ZAC12*-ZAC12*-GGGC and ZAC12*-ZTaq_3-GGGC, were produced, characterized and labelled with 99mTc as a commonly used radionuclide for SPECT imaging in clinics.**’’**

**Discussion: “**This resulted in improved monomeric affinity and a two-fold increase of tumour-to-blood ratio as compared to the patental ZAC12 construct**”**

**“**As control we used the parental AC12 molecule as monomer binding but in dimeric format with a Taq polymerase binding Affibody molecule to create a construct of the same size as the high affinity dimer but with parental affinity. The two monomeric constructs, AC12 and SYNT-179, have been compared previously (Oroujeni 2023).**”**

I assume that the ZTaq_3 is a non-binding affibody. This must be mentioned explicitly in the text as well as already in the introduction. This is an import fact to understand the story!

**Answer. Thank you for pointing at this! To clarify this point, we have included the following text to Introduction section of the revised version of the manuscript:** “The first variant, ZAC12*-ZAC12*-GGGC contained two units of the parental ZAC12* Affibody molecule binding to B7-H3. To evaluate impact of binding, the second variant contained only one B7-H3-binding unit and the second unit was ZTaq_3 Affibody molecule, which is a nonbinding Affibody. ZTaq is an Affibody originally selected as a binder to Thermus aquaticus (Taq) DNA polymerase, which doesn’t interact with any mammalian proteins. Thus, ZAC12*-ZTaq_3-GGGC is a monovalent bi-Affibody.”

I also suggest to evaluate the specific uptake in vivo by blocking experiments or with non-binding similar affibodies conjugates. Using different cancer cell lines can result in different tumor properties (e.g. different blood flow, different necrosis etc.). Here we see a significantly different weight of the tumors of 0.34±0.18 g and 0.8±0.4 g.

**Answer. Thank you for pointing out this important aspect! To address this comment, we evaluated the specific uptake in vivo by blocking experiment. The following texts and figures are included to the Materials and Methods and the Results sections of the revised version of the manuscript:** “To show in vivo binding specificity, two groups of mice bearing SKOV-3 xenografts were intraperitoneally injected with 600 µg of Affibody molecules 40 min before injection of the best binders, [^99m^Tc]Tc-ZAC12*-ZAC12*-GGGC and [^99m^Tc]Tc-SYNT-179. The biodistribution was measured 4 h after injection as described above. ”

“The results of blocking of B7-H3 using preinjcetion of Affibody molecule before injection of the [^99m^Tc]Tc-ZAC12*-ZAC12*-GGGC and [^99m^Tc]Tc-SYNT-179 in mice bearing SKOV-3 as B7-H3-positive xenografts showed that the tumour uptake for both radioconjugates reduced when B7-H3 is blocked by injection of Affibody molecule before injection the radiolabelled conjugate, confirming B7-H3-specific binding in vivo. ”

In Materials and Methods, the following text for the weight of mice and SKOV3 tumours were updated because of additional animal study.

‘’Biodistribution measurement was performed two weeks after cell implantation. Average animal weight was 16.517.9±2.11.5 g. Average tumour weight was 0.3427±0.18 g and 0.8±0.4 g for SKOV-3 and Ramos xenografts, respectively.’’

**We have added the results of this experiment as Fig. 9 to the Results section of the revised manuscript .** “Fig 9 Uptake of (A) [99mTc]Tc-ZAC12*-ZAC12*-GGGC and (B) [99mTc]SYNT-179 on SKOV-3 (B7-H3-positive) xenografts at 4 h after injection. B7-H3 was blocked with pre-injection of 600 µg of Affibody molecule 40 min before injection of the radioconjugate. Data are expressed as %ID/g and are averages from four mice±SD. Asterisk (*) marks significant (p<0.05) difference. ”
Introduction:

As already mentioned, it must be described that ZAC12*-ZTaq_3-GGGC is a monovalent bi-affibody.
Also important is to mention the affinity of SYNT-179!

**Answer. Thank you for pointing out this. The following texts and figures are included to the Materials and Methods and the Results sections of the revised manuscript:** “ Z_Taq_ is an Affibody originally selected as a binder to Thermus aquaticus (Taq) DNA polymerase, which doesn’t interact with any mammalian proteins. Thus, ZAC12*-ZTaq_3-GGGC is a monovalent bi-Affibody.”

The Affinity of SYNT-179 was added to the Table 2.

Material and methods:
Why there was not injected the same amount (mol) of affibodies in the mice.

**Answer. Thank you for pointing out this. The same mol was injected for all three variants. To clarify it better, the mass based on nmol was added to the text in the Materials and Methods (0.4 nmol).**

Please clarify with which affibody the target was blocked in the specific assay (Fig. 4). Why is the blocking for [99mTc]Tc-ZAC12*-ZTaq_3-GGGC only about 60%?

**Answer. Thank you for pointing out this important point! “There is a clear risk that a true, specific interaction is deemed nonspecific due to the blocked measurement not approaching zero in signal (incomplete “blocking”). The fact that 10, 100, or even 1000 times excess of unlabeled Ligand have been added to the labeled Ligand does not guarantee a completely blocked Target. The following reference provides more information about this assay:**

Björkelund H, Gedda L, Andersson K. Avoiding false negative results in specificity analysis of protein-protein interactions. J Mol Recognit. 2011 Jan-Feb;24(1):81-9.

Results:
An antiaffibody-ab was used to determine the amount of affibodies which bounds to the cell. I do not understand why only half affibody was detected for ZAC12*-ZTaq_3-GGGC. In my understanding I would expect this for SYNT-179 (only "one" affibody).???

**Answer. Thank you for pointing out this important point! The reviewer points out that the low affinity ZAC12*-ZTaq_3-GGGC seems to have a lower amplitude than the high affinity, affinity matured SYNT-179 monomer or the high affinity homodimeric ZAC12*-ZAC12-GGGC constructs. According to the current curve fit, the authors agree that the visual impression may lead in that direction. It is however difficult to judge if this is indeed the case, as the affinity is low and it is the last data point that suggests that the curve may reach saturation. With the poor affinity of this construct, it could be that even higher concentrations would have allowed the curve to reach a higher amplitude. The EC50 of the ZAC12*-ZTaq_3-GGGC is in the same range as that for ZAC12 monomer suggesting that the control works as it should, but that the detection either is hampered by ability of the secondary reagent to detect the ZTaq portion of the construct, or that it is due to improper curve fit and false conclusion of the amplitude. We added the following text in the discussion: "**On the other hand, the detection efficiency of secondary reagents to the heterodimeric Z-scaffold may have been impacted, which can be suggested from cell binding experiments in Fig 2 where the absolute signal seems to be lower than for the other constructs."

Table 2 stability of the monovalents are missing.

**Answer. Thank you for pointing out this! The data have been implemented in the revised version of the manuscript.**

I suggest to put results of Fig. 3 into a supplementary file.

**Answer. Thank you for pointing out this! The changes suggested by reviewer have been implemented in the revised version of the manuscript.** Supplemental Fig. 1. Characterization of anti-B7-H3 Affibody molecules. Reversed-phase HPLC chromatogram of non-labelled (green) and radiochromatogram (black) of 99mTc-labelled of (A) ZAC12*-ZAC12*-GGGC and (B) ZAC12*-ZTaq_3-GGGC Affibody molecules. The retention times (Rt) are expressed in minutes.

Table 3 is it really "salivary" or "salivary glands"

**Answer. Thank you for pointing out this! The change suggested by the reviewer has been implemented in the revised version of the manuscript.**

Table 3 and 4; Fig. 7 Please us for all biodistribution study results bar graphics and but the number in the supplementary file.

**Answer. Thank you for pointing at this! The changes suggested by reviewer have been implemented in the revised version of the manuscript. The following tables were added to Supplementary file.**

‘’Supplemental Table 1. Comparative biodistribution of ^99m^Tc-labelled Affibody molecules in BALB/C nu/nu mice bearing SKOV-3 xenografts 4 h after injection. Data are expressed as the percentage of administered activity (injected probe) per gram of tissue (% ID/g). The data are presented as the average (n = 4) and SD. Error bars might not be seen because they are smaller than the symbols.’’

‘’Supplemental Table 2. Tumour-to-organ ratios of 99mTc-labelled Affibody molecules in BALB/C nu/nu mice bearing SKOV-3 xenografts 4 h after injection. The data are presented as the average (n = 4) and SD; Supplemental Table 3. Biodistribution of ^99m^Tc-labelled Affibody molecules in BALB/C nu/nu mice bearing SKOV-3 (B7-H3 positive) and Ramos (B7-H3 negative) xenografts 4 h after injection. Data are expressed as the percentage of administered activity (injected probe) per gram of tissue (% ID/g). The data are presented as the average (n = 4) and SD. Error bars might not be seen because they are smaller than the symbols.’’

The following figures were added to the revised version of the manuscript: ‘’Fig. 5 Comparative biodistribution of ^99m^Tc-labelled Affibody molecules in BALB/C nu/nu mice bearing SKOV-3 xenografts 4 h after injection. Data are expressed as the percentage of administered activity (injected probe) per gram of tissue (% ID/g). The data are presented as the average (n = 4) and SD’’; ‘’Fig. 6 Tumour-to-organ ratios of ^99m^Tc-labelled Affibody molecules in BALB/C nu/nu mice bearing SKOV-3 xenografts 4 h after injection. The data are presented as the average (n = 4) and SD.’’

Fig. 6 Please use the same order as before!

**Answer. Thank you for pointing out this! The changes suggested by the reviewer have been implemented in the revised version of the manuscript. The Fig 6 was replaced by Fig 7.**

‘’Fig. 7 Imaging of (A) [^99m^Tc]Tc-SYNT-179, (B) [^99m^Tc]Tc-ZAC12*-ZAC12*-GGGC (C) [^99m^Tc]Tc-SYNT-179 in BALB/C nu/nu mice bearing B7-H3-positive SKOV-3 xenografts 4 h after injection. Linear relative scale (arbitrary units normalized to a maximum count rate) is provided for each image. Scales were adjusted to show red pixels in xenografts. Arrows point at tumour (T) and liver (L).’’

Discussion:
I do not understand the sentence: "this high-affinity interaction was relatively nonabundant". Please explain.

**Answer. Thank you for pointing out this! To clarify this comment, the revision on the following text was done.**

**"**The affinity maturation enabled to obtain lower K_D_ values for the strongest interaction of [^99m^Tc]Tc-SYNT-179 compared with both parental [^99m^Tc]Tc-ZAC12-GGGC and dimeric [99mTc]Tc-ZAC12*-ZAC12*-GGGC, but this high-affinity interaction was relatively less abundant (K_D1_=0.028±0.001nM, %weight_1_=12).The most abundant interaction of [^99m^Tc]Tc-SYNT-179 (K_D2_=8.2±0.5 nM, %weight_2_=88) was 8-fold stronger that the most abundant interaction of [^99m^Tc]Tc-ZAC12-GGGC (K_D2_=68.8±7.4, %weight_2_=67). **"**

Reviewer #3: The authors present a study on 2 Affibody dimers that target B7-H3 and compared to the affinity matured SYNT-179 affibody. The conjugates were labeled with 99mTc and studied in vitro and in vivo using B7-H3-expressing SKOV-3 cells and B7-H3-negative cells (Ramos) as a negative control. Despite high (sub)nanomlar affinity to B7H3+ cells, in vivo results showed that tumour uptake and tumour-to-organ ratios for the new affibody dimer conjugate were inferior to the previously studied affinity matured SYNT-179. The authors conclude by giving valuable insight and discuss the future design considerations for small affinity molecules that may lead to improved imaging agents. The experimental methods are well described. The manuscript is overall well written, with some comments made below for improvement.
**Answer. Thank you for appreciation of our work!**
Pg 2 line 39 - missing right bracket before [99mTc]-

Definitions of some acronyms missing (e.g., DTT)-

Pg 10 line 21 - remove 'is' from the sentence-

**Answer. Thanks. The corrections have been implemented in the revised version of the manuscript.**

Fig 1 - what is the significance of the bolded amino acids on the sequence? Also, it would be helpful to label (or provide the chemical structure) of the amino acids which are used to coordinate to 99mTc (assuming this was the GGGC based on prior works cited, this wasn't explicitly discussed or mentioned in this manuscript, would be worth to mention)-

**Answer. Thank you for pointing out this! To address this comment, the following texts were added to the revised version of the manuscript:**

‘’Fig. 1 (A) Amino acid sequences of the evaluated Affibody molecules and (B) general structure of the N3S chelators formed by a C-terminal cysteine and three adjacent amino acids. X1, X2 and X3 denote the side chains of the amino acids (in this case X1,X2, X3=hydrogen atom for glycine). The variable amino acids are marked in red and the chelator composition in green bold.’’

**To the introduction section:**‘’In this study, according to previous studies (Altai et al. 2012; Oroujeni et al. 2021), peptide-based cysteine containing chelator –GGGC was incorporated at the C-terminus at Affibody molecules for labelling with 99mTc. In this complex (Ahlgren et al. 2009, Altai et al. 2012), the amide nitrogens of amino acids from glycine and the thiol group of cysteine at peptide-based cysteine containing chelator form together a N3S chelator, providing a stable complex with 99mTc (Figure 1B).’’ **To the Result section:** ‘’To evaluate targeting properties of Affibody molecule labelled with 99mTc, the peptide-based cysteine containing –GGGC chelator was incorporated at the C-terminus of Affibody molecules. Fig. 1B shows the general structure of the N3S chelator formed by a C-terminal cysteine and three adjacent amino acids (triglycine-cysteine, ̶ GGGC, chelator). ‘’

Pg 13 line 3 - missing 'e' on end of molecule-

Pg 13 line 45 - remove extra "." after bracket-

Pg 15 line 1 - "both 99mTc-labelled conjugates were…"

**Answer. Thank you for pointing out this!** **The corrections suggested by the reviewer have been implemented in the revised version of the manuscript.**

Fig 3 - it would be better to stack the radiotrace and UV trace of each pair of chromatograms so a more direct comparison can be made.

**Answer. Thank you for pointing out this! The both radio and UV chromatograms were stacked and Fig 3 was transferred to Supplemental Fig 1.**’’ Supplemental Fig. 1. Characterization of anti-B7-H3 Affibody molecules. Reversed-phase HPLC chromatogram of non-labelled (green) and radiochromatogram (black) of 99mTc-labelled of (A) ZAC12*-ZAC12*-GGGC and (B) ZAC12*-ZTaq_3-GGGC Affibody molecules. The retention times (Rt) are expressed in minutes.’’

Table 3 and Table 4 - consider visualizing this data in bar graphs, and move the tabulated data to the SI-

**Answer. Thank you for pointing out this! The changes suggested by the reviewer have been implemented in the revised version of the manuscript.**

‘’Supplemental Table 1. Comparative biodistribution of ^99m^Tc-labelled Affibody molecules in BALB/C nu/nu mice bearing SKOV-3 xenografts 4 h after injection. Data are expressed as the percentage of administered activity (injected probe) per gram of tissue (% ID/g). The data are presented as the average (n = 4) and SD. Error bars might not be seen because they are smaller than the symbols.’’

‘’Supplemental Table 2. Tumour-to-organ ratios of 99mTc-labelled Affibody molecules in BALB/C nu/nu mice bearing SKOV-3 xenografts 4 h after injection. The data are presented as the average (n = 4) and SD; Supplemental Table 3. Biodistribution of ^99m^Tc-labelled Affibody molecules in BALB/C nu/nu mice bearing SKOV-3 (B7-H3 positive) and Ramos (B7-H3 negative) xenografts 4 h after injection. Data are expressed as the percentage of administered activity (injected probe) per gram of tissue (% ID/g). The data are presented as the average (n = 4) and SD. Error bars might not be seen because they are smaller than the symbols.’’

The following figures were added to the revised version of the manuscript: ‘’Fig. 5 Comparative biodistribution of ^99m^Tc-labelled Affibody molecules in BALB/C nu/nu mice bearing SKOV-3 xenografts 4 h after injection. Data are expressed as the percentage of administered activity (injected probe) per gram of tissue (% ID/g). The data are presented as the average (n = 4) and SD.’’; ‘’Fig. 6 Tumour-to-organ ratios of ^99m^Tc-labelled Affibody molecules in BALB/C nu/nu mice bearing SKOV-3 xenografts 4 h after injection. The data are presented as the average (n = 4) and SD.’’

Fig 6 - should be replotted. It looks like a screen shot of the image visualization analysis software. What is the bar graph on the left of the image? This should show the values for the scale bar (min and maximum in %ID/g or SUV, whatever the convention is in your field) and the images should be maximum intensity projections (MIP) slices.

**Answer. Thank you for pointing out this!** **The corrections suggested by the reviewer have been implemented in the revised version of the manuscript.** Fig 6 was replaced by Fig 7. ‘’Fig. 7 Imaging of (A) [99mTc]Tc-SYNT-179, (B) [99mTc]Tc-ZAC12*-ZAC12*-GGGC (CB) [99mTc]Tc-ZAC12*-ZTaq_3-GGGC and (C) [99mTc]Tc-SYNT-179 in BALB/C nu/nu mice bearing B7-H3-positive SKOV-3 xenografts 4 h after injection. Linear relative scale (arbitrary units normalized to a maximum count rate) is provided for each image. Scales were adjusted to show red pixels in xenografts. Arrows point at tumour (T) and liver (L).’’

Pg 20 line 56 - why was the tumour uptake in B7-H3-positive SKOV-3 and B7-H3-negative Ramos xenografts for the 'Taq' affibody not shown? Consider including this plot in the SI, even if the results are not significant or positive.

**Answer. Thank you for pointing out this! To address this comment, the data for [99mTc]Tc-ZAC12*-ZTaq_3-GGGC were added to Fig 8 and Supplemental Table 3.**

‘’Fig. 8 Uptake of (A) [99mTc]Tc-ZAC12*-ZAC12*-GGGC and (B) [99mTc]Tc-ZAC12*-ZTaq_3-GGGC on SKOV-3 (B7-H3-positive) and Ramos (B7-H3-negative) xenografts at 4 h after injection. Data are expressed as %ID/g and are averages from four mice±SD. Asterisk (*) marks significant (p<0.05) difference;’’

‘’Supplemental Table 3. Biodistribution of 99mTc-labelled Affibody molecules in BALB/C nu/nu mice bearing SKOV-3 (B7-H3 positive) and Ramos (B7-H3 negative) xenografts 4 h after injection. Data are expressed as the percentage of administered activity (injected probe) per gram of tissue (% ID/g). The data are presented as the average (n = 4) and SD.’’

**The following text in the Results section was revised in the revised version of the manuscript:**
‘’To test in vivo specificity of [99mTc]Tc-ZAC12*-ZAC12*-GGGC and [99mTc]Tc-ZAC12*-ZTaq_3-GGGC , the tumour accumulation in B7-H3-positive SKOV-3 xenografts and in B7-H3-negative Ramos xenografts was compared (Fig. 87 and Supplemental Table 3). The uptake of [99mTc]Tc-ZAC12*-ZAC12*-GGGC in SKOV-3 xenografts was significantly (p < 0.005) higher than in Ramos. This shows that the tumour accumulation was dependent on expression of B7-H3. No significant difference in tumour uptake in between B7-H3-positive SKOV-3 and B7-H3-negative Ramos xenografts for [99mTc]Tc-ZAC12*-ZTaq_3-GGGC was observed (Fig. 8B and Supplemental Table 3). ‘’

Fig 8- same comment as for Fig 6. Also, were the animals inoculated with both negative and positive xenografts? Or two sets of animals, one with positive tumours and the other with negative tumours only?

**Answer. Thank you for pointing out this!** **The comment suggested by the reviewer have been implemented in the revised version of the manuscript.**

‘’Fig. 10 Imaging of [99mTc]Tc-ZAC12*-ZAC12*-GGGC in BALB/C nu/nu mice bearing (A) B7-H3-negative Ramos xenografts and (B) B7-H3-positive SKOV3 xenografts 4 h after injection. 7.8 µg of labelled Affibody molecule (14 MBq, 100 µL in PBS) was injected into the tail vein. Arrows point at tumours (T). The animals were imaged simultaneously. Linear relative scale (arbitrary units normalized to a maximum count rate) was adjusted to visualize B7-H3-positive SKOV-3 xenografts.’’

We have simultaneously imaged 2 different mice, one implanted with SKOV-3 and the second with Ramos. The Ramos and SKOV3 xenografts are in 2 mice.

The authors refer to the imaging as 'nanoSPECT/CT' and at time 'microSPECT/CT'. is there a difference? If not, be consistent throughout.
**Answer. Thank you for pointing out this!** **The correction suggested by the reviewer have been implemented in the revised version of the manuscript.**

**Additional changes/corrections**

**1-Animal ethical permit is updated in ‘’2.5. In Vivo Studies.’’**

**‘**’All animal studies were approved by the Ethics Committee for Animal Research in Uppsala (Sweden), following the national legislation on the protection of laboratory animals (permit 5.8.18-00473/2021, approved 26 February 2021).**’’**

**2-Updated Funding**

**‘**’This research was funded by Swedish Cancer Society (Cancerfonden), grants number: 23 2645 PjF 01H (A.O.), CAN 21 1485 (V.T.), and the Swedish Research Council (Vetenskapsrådet), grant number 2022-00556 (A.O.); 2023-02158 (V.T.); Personal postdoctoral grant from Uppsala University (UFV-PA 2022/3323) (M.O.), Svenska Sällskapet för Medicinsk Forskning (815200-8317) (M.O.).**’’**
